# Targeting the RT loop of Src SH3 in Platelets Prevents Thrombosis without Compromising Hemostasis

**DOI:** 10.1002/advs.202103228

**Published:** 2022-01-12

**Authors:** Jianhua Mao, Kongkai Zhu, Zhangbiao Long, Huimin Zhang, Bing Xiao, Wenda Xi, Yun Wang, Jiansong Huang, Jingqiu Liu, Xiaofeng Shi, Hao Jiang, Tian Lu, Yi Wen, Naixia Zhang, Qian Meng, Hu Zhou, Zheng Ruan, Jin Wang, Cheng Luo, Xiaodong Xi

**Affiliations:** ^1^ State Key Laboratory of Medical Genomics Shanghai Institute of Hematology Collaborative Innovation Center of Hematology Ruijin Hospital Shanghai Jiao Tong University School of Medicine Shanghai 200025 China; ^2^ Drug Discovery and Design Center the Center for Chemical Biology State Key Laboratory of Drug Research Shanghai Institute of Materia Medica Chinese Academy of Sciences University of Chinese Academy of Sciences Shanghai 201203 China; ^3^ School of Life Science and Technology Shanghai Tech University Shanghai 201210 China; ^4^ Shanghai Institute of Hypertension Ruijin Hospital Shanghai Jiao Tong University School of Medicine Shanghai 200025 China; ^5^ School of Pharmaceutical Science and Technology Hangzhou Institute for Advanced Study UCAS Hangzhou 310024 China

**Keywords:** antithrombotic target, bleeding risk, E97A knock‐in mice, small molecule, *β*3/Src interaction

## Abstract

Conventional antiplatelet agents indiscriminately inhibit both thrombosis and hemostasis, and the increased bleeding risk thus hampers their use at more aggressive dosages to achieve adequate effect. Blocking integrin *α*IIb*β*3 outside‐in signaling by separating the *β*3/Src interaction, yet to be proven in vivo, may nonetheless resolve this dilemma. Identification of a specific druggable target for this strategy remains a fundamental challenge as Src SH3 is known to be responsible for binding to not only integrin *β*3 but also the proteins containing the PXXP motif. In vitro and in vivo mutational analyses show that the residues, especially E97, in the RT loop of Src SH3 are critical for interacting with *β*3. DCDBS84, a small molecule resulting from structure‐based virtual screening, is structurally validated to be directed toward the projected target. It specifically disrupts *β*3/Src interaction without affecting canonical PXXP binding and thus inhibits the outside‐in signaling‐regulated platelet functions. Treatment of mice with DCDBS84 causes a profound inhibition of thrombosis, equivalent to that induced by extremely high doses of *α*IIb*β*3 antagonist, but does not compromise primary hemostasis. Specific targets are revealed for a preferential inhibition of thrombosis that may lead to new classes of potent antithrombotics without hemorrhagic side effects.

## Graphical abstract

Patients receiving aggressive treatment with conventional antiplatelet agents are subject to the risk of bleeding. DCDBS84, a small molecule specifically targeting c‐Src SH3 RT‐loop around E97, achieves a potent antithrombotic effect without compromising primary hemostasis by selectively blocking outside‐in signaling through integrin *α*IIb*β*3. A new strategy for the development of antithrombotic therapies is provided here.

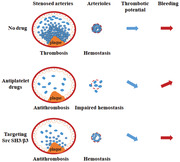

## 1 Introduction

Integrin *α*IIb*β*3 constitutes the final common pathway for various agonists in inducing platelet aggregation^[^ ^1^, ^2^ ^]^ which is essential for hemostasis to arrest bleeding and for thrombosis to occlude blood flow in arterial ischemia. Currently available antiplatelet (antithrombotic) agents such as aspirin, clopidogrel, and eptifibatide ultimately exert their effects by inhibiting platelet aggregation, specifically by preventing ligands from binding to the *α*IIb*β*3 receptor, and thus confront a basic issue: bleeding risk increases concurrently with enhancement of their antithrombotic effect.^[^ ^3^ ^]^ The bleeding side effect of *α*IIb*β*3 antagonists was also attributed to drug‐induced activating conformational changes in *α*IIb*β*3.^[^ ^4^ ^]^ Accordingly, targeting *α*IIb*β*3 signaling pathways instead of antagonizing the receptor has been thought to be an alternative therapeutic modality for treating thrombotic diseases.^[^ ^5^ ^]^

Indeed, a number of basic studies have shown that genetic manipulations, such as the Y747F and Y759F mutations or RGT deletion of integrin *β*3, Wiskott Aldrich syndrome protein, growth arrest‐specific protein 6 or calcium‐and integrin‐binding protein 1 deficiency, disrupting *α*IIb*β*3 outside‐in signaling result in a substantial inhibition of thrombotic potential without serious bleeding episodes.^[^ ^6^, ^7^, ^8^, ^9^, ^10^, ^11^ ^]^ These observations support a conclusion that the antithrombosis/hemostasis dilemma, the major disadvantage of receptor‐blocking strategies, can potentially be overcome by targeting outside‐in signaling. Despite these genetic insights, the present lack of well‐defined druggable targets that specifically regulate outside‐in signaling have hindered the translation of this concept into more effective and safer therapeutic approaches. In attempt to develop agents that specifically regulate outside‐in signaling, previous studies have shown that peptides targeting the Src/*β*3 interaction (myr‐RGT), the G*α*13/*β*3 interaction (myr‐FEEERA) and the 14‐3‐3*ζ*/*β*3 interaction (myr‐KEATSTF) inhibited the activation of c‐Src by blocking outside‐in signaling.^[^ ^12^, ^13^, ^14^ ^]^ Given the central role of the interaction of Src SH3 with integrin *β*3 cytoplasmic domain in outside‐in signaling,^[^ ^15^, ^16^ ^]^ dissociation of the Src/*β*3 interaction by targeting Src SH3 would be the strategy of choice for selectively regulating outside‐in signaling. However, there are thus far no in vivo data supporting that the antithrombotic effect without bleeding can be achieved by directly separating the Src/*β*3 interaction and a druggable target for this purpose remains to be identified.

In the context of Src activation by molecular interaction in various important biological processes, the Src SH3 binds proteins containing the PXXP motif.^[^ ^17^ ^]^ The highly conserved W118 in SH3 is thought to be essential for this canonical binding and thereby Src activation as evidenced by the decreased Src kinase activity caused by the mutation of this residue.^[^ ^18^ ^]^ In contrast to the “canonical” binding which is involved in a variety of regulatory pathways to activate Src, the *β*3/Src interaction is unique in that it does not require the PXXP motif. It was thus referred to as “noncanonical” binding in this study.

Motivated by the updated understanding of the molecular basis for the regulation of thrombosis and hemostasis, we aimed to identify the specific targets by experimentally distinguishing residues in Src SH3 that significantly contribute to its noncanonical and/or canonical binding. In an attempt to achieve a preferential inhibition of arterial thrombosis, the effect of small molecules targeting the noncanonical *β*3/Src interaction was examined in vitro and in vivo and its potential to facilitate the development of innovative and safer antithrombotic agents was assessed.

## 2 Results

### 2.1 Identification of the Antithrombotic Targets that do not Compromise Hemostasis

**Figure 1:**
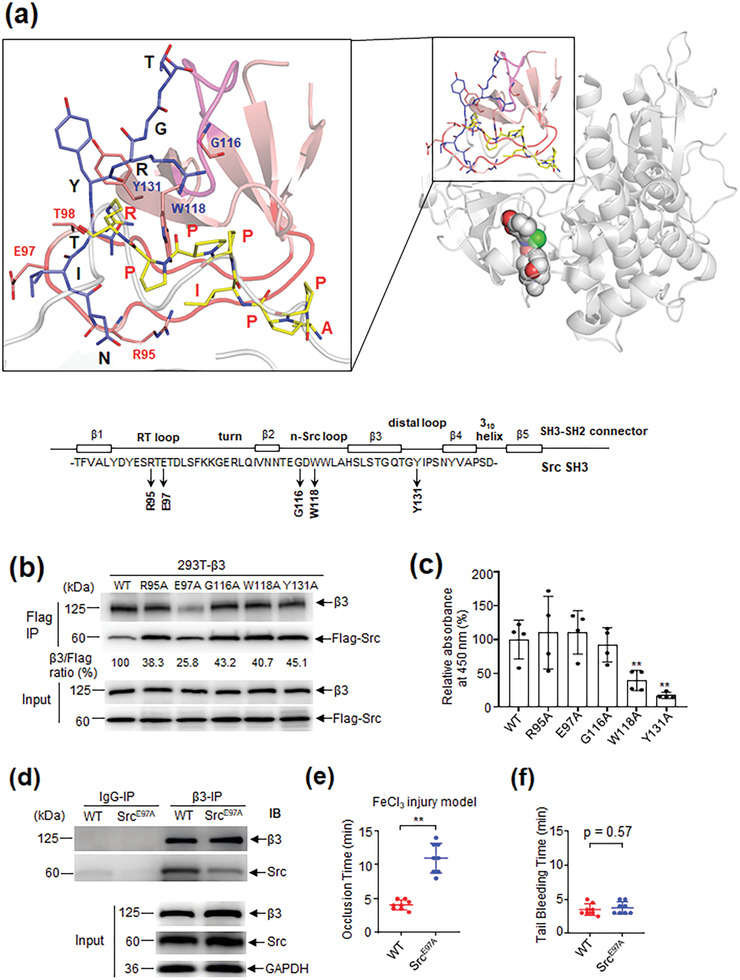
Identification of antithromobotic targets based on the interaction of Src SH3 with integrin *β*3 that do not compromise hemostasis. a) The comparison of binding modes results of *β*3 heptapeptide (NITYRGT) and a class II peptide APPIPPPR with SH3 domain and the schematic representation of the structure of Src SH3 domain. The full length Src structure (PDB code: 2H8H)^[^ ^52^ ^]^ was shown as cartoon with grayish‐white color while its SH3 domain was colored in salmon. RT loop and n‐Src loop of SH3 were colored in red and magenta respectively. Peptides and the interested residues in the top panel were shown as sticks with different colors. The carbon atoms in heptapeptide and APPIPPPR peptide were colored in light blue and yellow, respectively. The binding mode of heptapeptide with SH3 were obtained by molecular docking using Glide program (Schrödinger, LLC, New York, NY, 2015), and the binding mode of APPIPPPR with SH3 were obtained from X‐ray crystal structure with PDB code 4QT7.^[^ ^19^ ^]^ Down panel is the schematic representation of the structure of Src SH3 domain and the positions of the mutant amino acids. b) Co‐IP results show the interaction of integrin *β*3 with WT or mutant (R95A, E97A, G116A, W118A, and Y131A) Src in 293T cells. c) ELISA results show the amino acids in Src SH3 critical for binding to the RLP1 peptide containing the canonical PXXP motif. The wells of 96‐well plate were coated with the Flag antibody and incubated sequentially with the purified WT or mutant (R95A, E97A, G116A, W118A, and Y131A) Src with a Flag tag, biotin‐RLP1 peptide and HRP‐conjugated streptavidin. The binding intensity of the biotin‐RLP1 was then measured. Data were presented as mean ± SD. Pairwise comparisons were performed using the Student's *t*‐test and two‐tailed analysis by using GraphPad Prism. (*n* = 4, **p < 0.01). d) Co‐IP assays show the interaction between *β*3 and Src in platelets from WT and Src^E97A^ transgenic mice. e) FeCl_3_‐induced carotid arterial thrombus formation of WT and Src^E97A^ transgenic mice. Data were presented as mean ± SD. Student's *t*‐test with two‐tailed analysis was performed by using GraphPad Prism. (*n* = 8, **p < 0.01). f) Tail bleeding time of WT and Src^E97A^ transgenic mice (*n* = 8). Data were presented as mean ± SD. Student's *t*‐test with two‐tailed analysis was performed by using GraphPad Prism.

The comparison of the binding modes of *β*3 heptapeptide (NITYRGT) with SH3 obtained by molecular docking and the peptide APPIPPPR with SH3 originated from crystal structure (PDB code: 4QT7)^[^ ^19^ ^]^ indicated that the PXXP peptides and the C‐terminal peptide of *β*3 bind to SH3 in different orientations and the binding site for NITYRGT extends from the n‐Src loop into the RT loop, whereas the PXXP motif that adopts a polyproline type II (PPII) helix binds to the structures between the RT loop and n‐Src loop (**Figure** **1**a). These results are consistent with previous studies.^[^ ^20^, ^21^ ^]^

The proximity of the N‐terminus of the *β*3 heptapeptide to the RT loop suggested that the residues around E97 apparently participate in noncanonical binding. We thus felt it necessary to identify which residues in SH3 involve in noncanonical versus canonical binding. Such precise structural information delineating these two distinct molecular interactions should effectively facilitate the design of therapeutic agents which specifically block outside‐in signaling.

Recalling that residues (R95 and E97) in Src SH3 RT loop were implicated in interaction with the *β*3 cytoplasmic tail or its C‐terminal heptapeptide,^[^ ^21^ ^]^ we conducted alanine mutational screening of these residues. We also mutated two residues (G116 and W118) in SH3's n‐Src loop, as well as the Y131 which was implicated in canonical binding. The interaction of the R95‐ or E97‐mutated Src SH3 with *β*3 but not that with the RLP1 (RKLPPRPSK, PXXP‐containing) peptide^[^ ^20^ ^]^ was significantly reduced, whereas the interaction of the G116‐, W118‐ and Y131‐mutants with RLP1 peptide but not that with *β*3 was interfered (Figure 1b,c). These results indicate that the residues of the RT loop positioned near E97 were primarily responsible for noncanonical binding whereas residues near W118 in the n‐Src loop contribute to its canonical binding. Mutagenesis results support that the RT loop of Src SH3 is required for its interaction with *β*3, and implicate E97 as a key residue mediating this interaction.

To verify these data in vivo, a Src^E97A^ knock‐in mouse model was established. The *β*3/Src interaction was disrupted in these mice (Figure 1d). Moreover, the Src^E97A^ mice exhibited in FeCl_3_ thrombosis model a significantly reduced thrombotic potential (Figure 1e). Notably, the Src^E97A^ mice had an unaffected bleeding time (Figure 1f).

### 2.2 Small Molecule DCDBS84 Specifically Binds to the RT Loop of Src SH3

**Figure 2:**
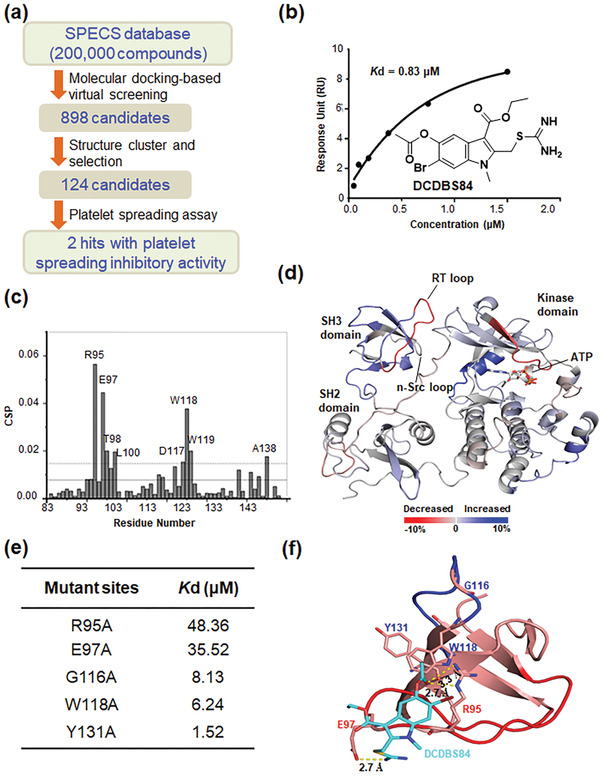
Small molecule DCDBS84 specifically binds to the RT loop of Src SH3. a) The workflow of screening for small molecules targeting SH3. b) Structural formula of DCDBS84. The dissociation constant (*K*d) of DCDBS84 binding to Src SH3 was calculated by SPR. c) Chemical shift perturbation (CSP) analysis of Src SH3 upon the binding of DCDBS84. Mean (solid line) and mean + SD (standard deviation) (dashed line) values were indicated. Residues with CSP values over the dashed line were labeled. d) Src/DCDBS84 interaction characterized by HDX‐MS. Percentage change of deuterium uptake at 60 min was mapped onto the crystal models of Src (PDB code: 2SRC),^[^ ^56^ ^]^ deuterium uptake was labeled as the color bar indicated. e) The *K*d of the DCDBS84 binding to purified mutant (R95A, E97A, G116A, W118A, and Y131A) Src SH3. f) The proposed binding mode of DCDBS84 with SH3 domain. The SH3 domain and DCDBS84 were shown as cartoon and sticks, respectively. The interacting residues were also shown as sticks. DCDBS84 was colored cyan. The RT loop and n‐Src loop of SH3 were colored red and blue respectively. The yellow dash lines indicated H‐bond interaction.

We employed structure‐based docking approaches by targeting the E97 site of SH3 to identify small molecules. A total of 124 compounds out of the 200 000 docked compounds of the SPECS database were selected (**Figure** **2**a). Platelet spreading assays revealed that small molecule No. 84 exerted a substantial inhibitory effect at a low concentration (31.25 µmol L^−1^), compared with others (Figure S1a, Supporting Information). No. 84, henceforth termed DCDBS84, was thus chosen as the candidate small molecule.

Surface plasmon resonance (SPR) analysis showed that the *K*d of DCDBS84 for SH3 was 0.83 µmol L^−1^ (Figure 2b; Figure S1b, Supporting Information). In nuclear magnetic resonance (NMR), DCDBS84 was titrated against ^15^N‐labeled SH3, and we detected significant chemical shift differences for residues R95, E97, T98, L100, D117, W118, W119, and A138 (Figure 2c; Figure S2a,b, Supporting Information), which were consistent with previous results with the *β*3 cytoplasmic tail and the C‐terminal heptapeptide (NITYRGT).^[^ ^21^ ^]^

Hydrogen‐deuterium exchange mass spectrometry (HDX‐MS) has often been applied to probe changes in protein dynamics induced by ligand interaction.^[^ ^22^, ^23^ ^]^ The different deuterium uptake (hydrogen/deuterium exchange) rates of peptides lead to the changes of the molecular mass of the targeted peptides, which can be detected by mass spectrometry. Hydrogen/deuterium exchange occurring upon ligand binding reveals the specific regions that interact with ligand as well as conformational changes induced by ligand binding. Herein, significant changes of the deuterium exchange rate were observed around the SH3 domain upon DCDBS84 combination (Figure 2d). Furthermore, analysis of disturbance of the SH3 peptides caused by the compound combination showed that the most substantial reduction in deuterium uptake (>5% reduction from apo) occurred with a peptide in the RT loop (89‐98) (Figure S2c,d, Supporting Information).

We also conducted SPR experiments to detect the binding of DCDBS84 or RLP1 peptide with SH3 mutant variants including R95A, E97A, G116A, W118A, and Y131A. The significantly increased *K*d values for the R95A and E97A variants (Figure 2e; Figure S3, Supporting Information) suggested that DCDBS84 preferentially binds at the SH3 RT loop in the vicinity of E97. In contrast, the G116A, W118A, and Y131A substitutions markedly affected RLP1 binding to SH3 (Figure S4, Supporting Information). Moreover, DCDBS84 did not inhibit the RLP1 peptide binding to SH3 (Figure S5a, Supporting Information).

Binding mode analysis of DCDBS84 with SH3 obtained by molecular docking again supported that DCDBS84 engages with the E97 residue positioned in the RT loop via H‐bond interaction (Figure 2f). Besides E97, the R95 and W118 residues respectively displayed H‐bond interaction and hydrophobic interaction with DCDBS84 (Figure 2f). Data suggest that DCDBS84 rightly simulated the integrin *β*3 cytoplasmic tail in terms of binding to Src SH3 via a site preferentially composed of E97 and the adjacent residues in the RT loop.

We then tested whether DCDBS84 is capable of directly influencing Src kinase activity. An in vitro kinase assay showed that DCDBS84 did not affect the tyrosine kinase activity of Src while PP2 (Src family kinase inhibitor) and dasatinib (tyrosine kinase inhibitor) did (Figure S5b, Supporting Information).

### 2.3 DCDBS84 Disrupts the Interaction of Integrin *β*3 with Src SH3

**Figure 3:**
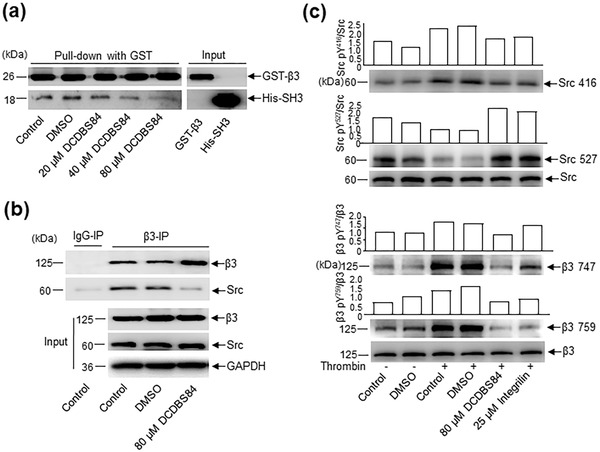
Small molecule DCDBS84 disrupts the interaction of integrin *β*3 with Src SH3, affecting the phosphorylation of *β*3 and Src. a) Combination between purified GST‐integrin *β*3 cytoplasmic tail fusion protein and purified His‐Src‐SH3 in the presence of DCDBS84 at different concentrations (20, 40, 80 µmol L^−1^), dimethylsulfoxide (DMSO) or control (without treatment of DCDBS84 or DMSO), was detected by a pull‐down assay. b) Platelet lysates were incubated with 80 µmol L^−1^ DCDBS84, DMSO or control (without treatment of DCDBS84 or DMSO), immunoprecipitated by anti‐integrin *β*3 antibody and immunoblotted with anti‐Src antibody. c) Platelets from volunteers were treated with or without thrombin (0.1 U µL^−1^) and incubated with 80 µmol L^−1^ DCDBS84, 25 µmol L^−1^ integrilin (positive control), DMSO or control (without treatment of DCDBS84, integrilin or DMSO), protein lysates were immunoblotted with antibodies specific for *β*3, phosphorylated *β*3 (*β*3^747^ and *β*3^759^), Src and phosphorylated Src (Src^416^ and Src^527^).

DCDBS84 was tested for its ability to interfere with the *β*3/Src interaction and was shown to be dose‐dependently active in glutathione *S*‐transferase (GST) pull‐down and co‐immunoprecipitation (co‐IP) (**Figure** **3**a,b). However, it did not affect the association of *β*3 with kindlin 3 or G*α*13 (Figure S5c, Supporting Information), both involved in outside‐in signaling through different mechanisms.^[^ ^13^, ^24^ ^]^ Characterized by the dephosphorylation of Tyr^527^ and the phosphorylation of Tyr^416^, the activation of Src is regulated by different signaling pathways. One of these has been well defined as outside‐in signaling‐dependent via association with *β*3 cytoplasmic tail.^[^ ^25^ ^]^ Other pathways are regulated by the proteins binding to Src SH3 through the canonical PXXP motifs.^[^ ^26^ ^]^

The effect of DCDBS84 on outside‐in signaling‐induced Src activation was further examined. In thrombin‐stimulated platelets, pretreatment with DCDBS84 resulted in a decrease of the phosphorylation of Src at Tyr^416^ and an increase of that at Tyr^527^ (Figure 3c). In addition, in view of the phosphorylation of *β*3 at Tyr^747^ and Tyr^759^ as the typical consequence of outside‐in signaling‐regulated Src activation,^[^ ^27^ ^]^ the effect of DCDBS84 on this was thus examined. Western blot (WB) data using specific antibodies show that the phosphorylation of Tyr^747^ and Tyr^759^ in response to thrombin were diminished by DCDBS84 treatment (Figure 3c) suggesting an impaired outside‐in signaling.

### 2.4 DCDBS84 Selectively Inhibits Platelet Functions Mediated by Outside‐In Signaling

**Figure 4:**
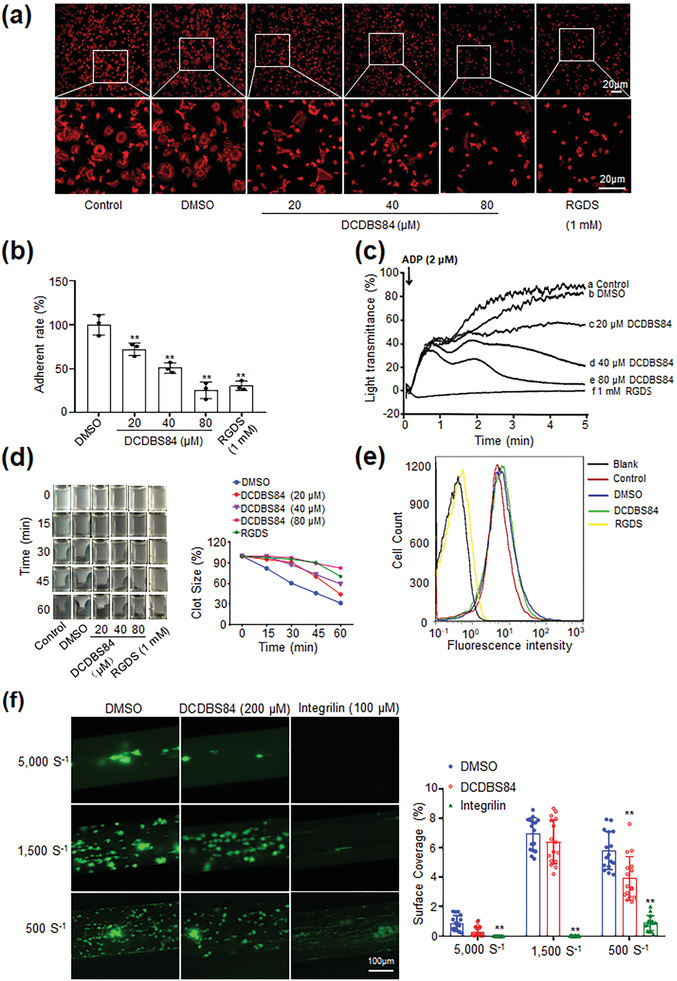
DCDBS84 selectively inhibits platelet functions mediated by outside‐in signaling. a) Effects of DCDBS84 on platelet spreading on immobilized fibrinogen at different concentrations (20, 40, 80 µmol L^−1^), DMSO, 1 mmol L^−1^ RGDS or control (without treatment of DCDBS84, RGDS, or DMSO). Scale bar is 20 µm. b) Effects of DCDBS84 on platelet stable adhesion on immobilized fibrinogen at different concentrations (20, 40, 80 µmol L^−1^), data shown in the pattern (mean ± SD) were derived from the results of three separate experiments (**p < 0.01). Student's *t*‐test with two‐tailed analysis was performed by using GraphPad Prism. c) Effects of DCDBS84 at different concentrations (20, 40, 80 µmol L^−1^), DMSO, 1 mmol L^−1^ RGDS or control (without treatment of DCDBS84, RGDS, or DMSO) on platelet aggregation stimulated by ADP (2 µmol L^−1^). d) Effects of DCDBS84 on fibrin clot retraction at different concentrations (20, 40, 80 µmol L^−1^), DMSO, 1 mmol L^−1^ RGDS or control (without treatment of DCDBS84, RGDS or DMSO). The left panel displayed photographs of the fibrin clots at every 15 min after initiated by 1 U mL^−1^ thrombin. The right panel analyzed the clot size rate (compared to the primary clot size) at different time points in each group. e) Effects of DCDBS84 on soluble fibrinogen binding to platelets. Data of representative photo show fibrinogen binding to platelets after treated with DMSO, DCDBS84 (80 µmol L^−1^), and RGDS peptide (1 mmol L^−1^) or control (without treatment of DCDBS84, RGDS, or DMSO). The blank was assessed using platelets treated without ADP. f) Effects of DCDBS84 on thrombus formation of human platelets at different shear rates. Hirudin‐anticoagulated whole blood was incubated with DMSO, DCDBS84 (200 µmol L^−1^) or integrilin (100 µmol L^−1^) prior to perfusion, and then perfused at different wall shear rates (5000, 1500, or 500 s^–1^) for 5 min. The thrombus formation was observed and imaged under an inverted fluorescent microscope. The left panel displayed the representative images to show platelet thrombi (scale bar is 100 µm). The right panel is the quantitative data to show the percentage of surface coverage. Data were presented as the mean ± SD deviation from 16 randomly selected visual fields of at least three independent experiments. One‐way analysis of variance (ANOVA) was adopted with normally distributed data assuming equal variances by using SPSS software. *p < 0.05, **p < 0.01, compared with DMSO treatment.

DCDBS84 dose‐dependently inhibited human platelet adhering and spreading on immobilized fibrinogen (**Figure** **4**a,b). In the presence of DCDBS84, ADP and thrombin‐induced irreversible platelet aggregation (Figure 4c; Figure S5d, Supporting Information) and fibrin clot retraction were inhibited (Figure 4d) while soluble fibrinogen binding remained unaffected (Figure 4e).

Then, in vitro platelet thrombus formation under flow conditions was examined. Results showed that at a wall shear rate of 5000 s^–1^, representing that in stenosed arteries, fewer, smaller, and thinner thrombi were observed with DCDBS84‐treated blood indicating an impaired thrombus growth at stenosed arteries and thereby an effective antithrombotic potential. At the shear rate of 1500 s^–1^, representing that in peripheral arterioles where bleeding usually occurs, the thrombus formation with DCDBS84‐treated blood was almost similar to that with controls in contrast to a drastic inhibition caused by *α*IIb*β*3 antagonist integrilin (Figure 4f). These results suggested that DCDBS84 specifically disrupted platelet outside‐in signaling.

### 2.5 DCDBS84 Effectively Suppresses Thrombus Formation without Prolonging Bleeding Time

**Figure 5:**
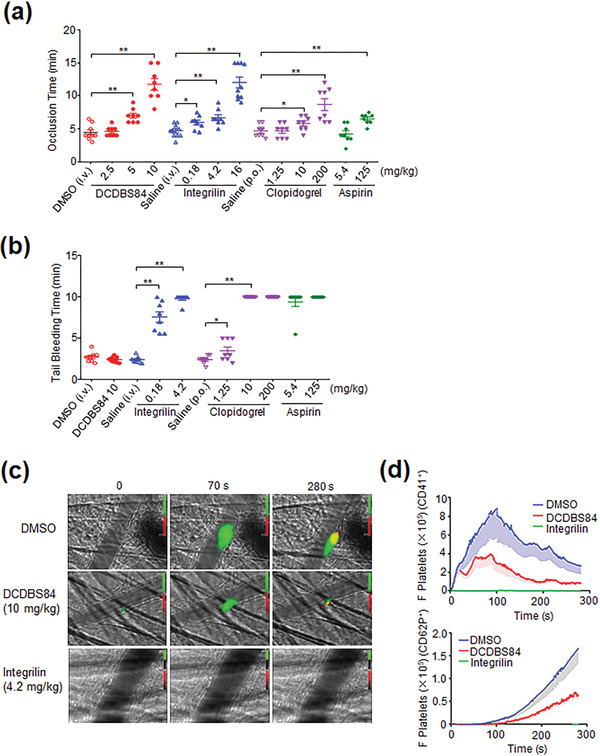
DCDBS84 effectively suppresses occlusive thrombus formation without affecting bleeding time. a) Quantitative analysis of FeCl_3_‐induced occlusive carotid artery thrombosis in mice when intravenously injected with DCDBS84 (dissolved in DMSO) at 2.5, 5, and 10 mg kg^−1^, integrilin (dissolved in saline) at 0.18, 4.2, and 16 mg kg^−1^), DMSO or saline. Intragastric administration of aspirin (5.4, 125 mg kg^−1^), clopidogrel (1.25, 10, 200 mg kg^−1^) or saline was performed at 24 and 2 h prior to the initiation of the carotid artery injury procedure. Data were shown as mean ± SD. Student's *t*‐test with two‐tailed analysis was performed by using GraphPad Prism. Sample size (*n*) was showed in each group, from 8 to 11, *p < 0.05 and **p < 0.01. To correlate the in vivo doses (mg kg^−1^) with the in vitro concentrations (molar concentration), the doses in mg kg^−1^ could be converted to molar concentrations based on the estimation of mouse blood volume, i.e., 72 mL kg^−1^ for a 6‐ to 8‐week mouse^[^ ^60^ ^]^ (70 mL kg^−1^ for a human adult).^[^ ^61^ ^]^ The estimated molar concentration is thus 68.1 µmol L^−1^ for 2.5 mg kg^−1^ DCDBS84 (509.21 Da) and 3 µmol L^−1^ for 0.18 mg kg^−1^ integrilin (831.96 Da), and so on. b) Tail bleeding time when the mice treated with DCDBS84 (10 mg kg^−1^), integrilin (0.18, 4.2 mg kg^−1^), DMSO, aspirin (5.4, 125 mg kg^−1^), clopidogrel (1.25, 10, 200 mg kg^−1^) or saline. Data were presented as mean ± SD. Student's *t*‐test with two‐tailed analysis was performed by using GraphPad Prism (*n* = 8, *p < 0.05 and **p < 0.01). c) The laser‐induced cremaster vessel injury model to monitor the thrombus formation in vivo. The mice were injected the Alexa‐fluor 488‐labeled CD41 and Alexa‐fluor 647‐labeled CD62P (P‐selectin) antibodies after anesthesia. Then the mice were treated with DMSO, DCDBS84 (10 mg kg^−1^) or integrilin (4.2 mg kg^−1^) for 30 min. Real‐time thrombus formation was monitored by video recording under a fluorescent microscope after the cremaster vessel was injured by laser. The green represents the platelets and the red represents the activated platelets, respectively. Representative images at 0, 70, and 280 s after injury are shown. d) Quantification of the data of (c). The median integrated platelet fluorescence (F Platelets) during thrombosis at 30 injury sites in 3 mice. Top panel is the CD41 fluorescent intensity and lower panel is the CD62P (P‐selectin) fluorescent intensity. The area under curve represents integrated platelet fluorescence and the dash area shows the standard deviation.

The in vivo effects of DCDBS84 were further examined in comparison with three conventional antithrombotic agents, including aspirin (cyclooxygenase inhibitor), clopidogrel (P2Y12 antagonist), and integrilin (*α*IIb*β*3 antagonist) at maintenance doses, loading doses after percutaneous coronary intervention (PCI), and extremely high doses that were about 20‐fold higher than the doses after PCI. PCI is clinically applied to relieve symptoms in patients with ischemic heart disease by placing stents into the stenosed coronary arteries. Antiplatelet agents are administered before, during and after PCI in order to minimize the risk of procedural ischemic complications such as myocardial infarction, stent thrombosis, and various degrees of myonecrosis.^[^ ^28^ ^]^ FeCl_3_‐induced occlusive carotid artery thrombosis model showed that DCDBS84 dose‐dependently prolonged the occlusion time starting at 2.5 mg kg^−1^, and the inhibition was further strengthened along with the increasing doses until to a drastic level at 10 mg kg^−1^ (**Figure** **5**a). Of note, integrilin at 0.18 mg kg^−1^ led to a relatively modest inhibitory effect which became greater at 4.2^[^ ^13^ ^]^ and 16 mg kg^−1^ (Figure 5a; Figure S6a, Supporting Information) indicating that the conventional dose of integrilin (0.18 mg kg^−1^ after PCI) is much lower than required to achieve its full antithrombotic potential. Aspirin hardly prolonged the occlusion time at 5.4 mg kg^−1^ (equivalent to 325 mg loading dose after PCI)^[^ ^28^ ^]^ and exhibited a weak inhibitory effect at an extremely high dose (125 mg kg^−1^) (Figure 5a). Clopidogrel had no effect at 1.25 mg kg^−1^ (≅ to 75 mg daily maintenance dose),^[^ ^28^ ^]^ and became active at 10 mg kg^−1^ (equivalent to 600 mg loading dose after PCI).^[^ ^28^ ^]^ A more significant inhibitory effect was achieved at an extremely high dose (200 mg kg^−1^) (Figure 5a). All these results clearly indicate that, in the in vivo thrombosis model employed in this study, the conventional doses of aspirin, clopidogrel and integrilin are inadequate in terms of their full antithrombotic potential. Importantly, when tail bleeding time was measured to evaluate the hemostatic potential, the results of DCDBS84‐treated mice, even at doses as high as 10 mg kg^−1^ that was equivalent to 16 mg kg^−1^ of integrilin in terms of inhibitory effect on thrombosis, were comparable to those of normal controls. In stark contrast to this scenario, the aspirin, clopidogrel and integrilin‐treated animals suffered from excessive bleeding even at 5.4, 10, and 0.18 mg kg^−1^ that became worse with the increasing doses until massive bleeding at 125, 200, and 4.2 mg kg^−1^ (Figure 5b; Figure S6b, Supporting Information), respectively. These data support a conclusion that because of the risk of bleeding, conventional antithrombotics such as aspirin, clopidogrel, and integrilin are commonly used at a dosage level corresponding to a modest effect and further suggest that the agents which do not compromise hemostasis such as DCDBS84 might be applied at aggressive doses to ensure an adequate prevention of thrombosis.

**Figure 6:**
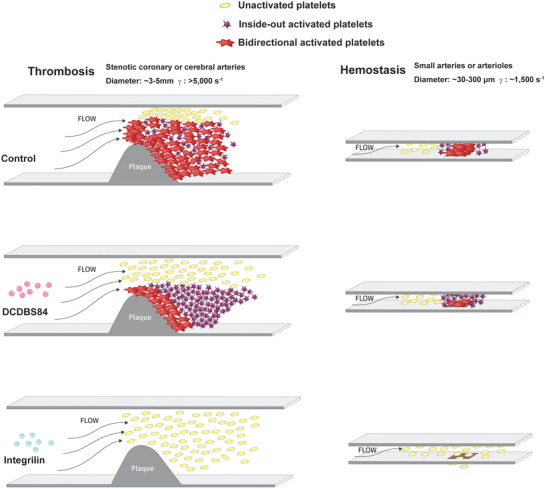
Working model to show the effect of DCDBS84 on thrombosis without compromising hemostasis.

The laser‐induced cremaster vessel injury model was employed to enable real‐time monitoring of in vivo thrombus formation. Different from a stable growth of thrombi at the vessel injury site composed of large amounts of activated platelets in normal controls or very little platelet adhesion and aggregation in integrilin‐treated animals, DCDBS84 treatment resulted in a significantly reduced thrombus growth owing to the less‐activated platelets (blocked outside‐in signaling) that would be less resistant to the impact of the increasing shear rates on thrombus formation. Of note, these platelets could still deposit a thinner layer at the site by virtue of their residual resistance (active inside‐out signaling) to weaker shear rates in contrast to the failure of thrombus formation with the integrilin‐treated mice (Figure 5c,d; Figure S6c,d, Supporting Information). The video data further showed that the platelet aggregates formed in DCDBS84‐treated animals looked loosely‐packed and less resistant to be detached by flow in contrast to the tightly packed and more stable aggregates in control animals (Movie S1–S3, Supporting Information). The effects of DCDBS84 on thrombosis and hemostasis are shown in **Figure** **6**.

## 3 Discussion

The present work focuses on the regulation of thrombosis and hemostasis, primarily involving the thrombus formation to impede or block blood flow. In the context of the formation of platelet thrombi, thrombosis usually refers to the pathological events that occur in arteries, coronary or cerebral arteries for example, and the physiological hemostasis tends to occur in peripheral vessels. Antiplatelet drugs are thus commonly used to treat thrombotic diseases even with bleeding as a major adverse effect since platelets are critical in both thrombosis and hemostasis. Clinical trials revealed that, at conventional doses, the occurrence of hemorrhagic and ischemic events seemed similar and greater platelet inhibition free of excessive bleeding was thus envisaged.^[^ ^29^, ^30^, ^31^ ^]^ It is generally accepted that the signal transduction through outside‐in and inside‐out directions in platelets is required for both thrombosis and hemostasis. In contrast to physiological hemostasis, where intact inside‐out signaling is able to maintain an adequate level of hemostatic potential, during thrombus formation in arteries, coronary arteries for example, outside‐in signaling is indispensable for the occlusive thrombosis.^[^ ^32^ ^]^ Preferential regulation of thrombosis over hemostasis may provide solutions and selectively blocking *α*IIb*β*3 outside‐in signaling has been proven as a promising strategy. To this end, Src SH3 seems an appropriate target since its binding to integrin *β*3 is central in transducing outside‐in signals.

In this study, we identified a novel antithrombotic target within Src SH3 that could effectively distinguish the “noncanonical” binding site for *β*3 cytoplasmic domain from the canonical binding site for PXXP motif and specifically regulate outside‐in signaling. Furthermore, a small molecule DCDBS84 directed to this target was selected and its effect on platelet function was further examined. The mechanisms for its potential to inhibit thrombosis without compromising hemostasis were analyzed especially in the context of different hydrodynamic conditions. Importantly in vivo, DCDBS84 exhibited a strong antithrombotic effect, comparable to that caused by high‐dose conventional antiplatelet drugs, without a hemorrhagic diathesis. This work illuminates feasible avenues for the development of safer antithrombotic agents.

To define specific antithrombotic targets within Src SH3, it was necessary to distinguish residues in SH3 that significantly contribute to its noncanonical and/or canonical binding, so as to minimize the impacts resulting from disruption of the biological processes that are regulated by canonical binding. Structural and mutational analyses showed that the particular residues in the RT loop especially the E97A significantly contributed to the interaction of Src SH3 with *β*3 (Figure 1b), suggesting that they might serve as the targets. The *β*3/Src interaction was significantly interrupted in the Src^E97A^ mouse model provided the first in vivo data (Figure 1d) showing that a markedly reduced thrombotic potential (Figure 1e) with normal bleeding time could be reached by disrupting *β*3/Src interaction (Figure 1f). These data indicate that the amino acids around E97 of the RT loop of Src SH3 specifically contributed to noncanonical *β*3 binding and thereby outside‐in signaling. The RT loop, especially E97, was thus validated as the candidate target for innovative antithrombotic strategies.

Virtual screening approach has been successfully applied to identify new scaffold ligands.^[^ ^33^, ^34^ ^]^ The basic idea for structure‐based virtual screening in this study was to mimic the structure of *β*3 cytoplasmic tail that specifically associates with the RT loop of Src SH3 especially E97 as the putative noncanonical binding. The screening strategy was based on crystallographic data with the PDB code 4HXJ^[^ ^35^ ^]^ together with the structural information regarding the *β*3/SH3 interaction (Figure 1a) and the RT loop of SH3. DCDBS84 was chosen out of 124 compounds resulted from virtual screening as the candidate for the functional research (Figure 2a and Figure S1a, Supporting Information).

To precisely define the targeting site for DCDBS84 in SH3, SPR, NMR, and HDX‐MS were employed. All the results reinforced the predominant contribution of the RT loop including E97 in DCDBS84 binding (Figure 2b,c; Figures S1b,c and S2c, Supporting Information).

Furthermore, mutational strategy was used to provide more specific information at the level of individual amino acids. SPR results indicated that DCDBS84 preferentially bound to the RT loop in the vicinity of E97 (noncanonical binding) but not W118 (known to be critical for canonical binding) (Figure 2e,f). Data further suggest that the small molecules selected by our strategy closely mimic the *β*3 cytoplasmic tail especially the NITYRGT peptide as evidenced by the similar chemical shift profiles of residues of SH3 in NMR.^[^ ^21^ ^]^ Furthermore, DCDBS84 affected neither the PXXP‐containing peptide binding to SH3 (Figure S5a, Supporting Information) nor the catalytic activity of Src (Figure S5b, Supporting Information). The characteristics of the molecular interaction, i.e., targeting the E97 in RT loop, conferred the specificity to DCDBS84 in regulating the signal transduction through *β*3 integrins.

DCDBS84 preferentially bound to the RT‐loop of Src SH3, different from RLP1 peptide containing the canonical PXXP motif which bound to SH3 predominantly requiring residues around the n‐Src loop. This allows a conclusion key to the prospect of future development that DCDBS84 could only affect the Src activation by “noncanonical” Src/*β*3 interaction but not that by canonical PXXP binding.

Src/*β*3 interaction is known to be essential in transducing outside‐in signals in platelets. While the other members of Src family kinases such as Lyn and Fgr might involve in integrin signaling,^[^ ^36^, ^37^ ^]^ they have not been shown to mediate outside‐in signaling in platelets by binding to the *β*3 tail which was proven thus far to be achieved only by Src. Spleen tyrosine kinase, downstream to and the substrate for Src, is important in the propagation of outside‐in signals in platelets by being recruited and activated upon Src activation.^[^ ^38^ ^]^ According to the mechanisms by which Src interacts with the *β*3 tail and the characteristics of the targeting site, DCDBS84 seemed unlikely to affect the above‐mentioned kinases in the context of outside‐in signaling via direct interaction with the *β*3 tail.

Protein tyrosine kinases (PTK), plays essential roles in different pathophysiological processes. PTK needs to be activated first that confers the catalytic activity to the kinase for functioning in downstream processes.^[^ ^39^, ^40^ ^]^ Tyrosine kinase inhibitors (TKi) have been developed to inhibit the activity of the activated PTK and block the downstream signal transduction by competing with ATP for the ATP binding site of PTK resulting in a reduction of tyrosine kinase phosphorylation of the substrates.^[^ ^41^ ^]^ Bruton's tyrosine kinase (BTK) is a nonreceptor tyrosine kinase and proven to be critical in the signal transduction of the B cell antigen receptor and other cell surface receptors.^[^ ^42^ ^]^ BTK inhibitor (BTKi), such as acalabrutinib, ibrutinib, and zanubrutinib, also inhibits the BTK activity via binding to the BTK kinase region.^[^ ^43^ ^]^ Totally different from TKi and BTKi, DCDBS84 inhibited the activation of Src but not its catalytic activity. This conclusion is based on the observations of the present study in which DCDBS84 specifically recognized the noncanonical binding site (Figure 2c–e; Figures S2 and S3, Supporting Information) and thereby dissociated the *β*3/Src interaction (Figure 3a,b). In addition, we provide evidence that DCDBS84 was unlikely able to suppress the catalytic activity of Src by showing that it did not affect the canonical binding site (Figure 2e; Figure S3, Supporting Information) and the activity of Src kinase in in vitro assays (Figure S5b, Supporting Information).

DCDBS84 was therefore supposed to be able to dissociate the *β*3/Src interaction by occupying the noncanonical binding site. Indeed, this interaction was unequivocally disrupted (Figure 3a,b). Results further showed an inhibitory effect of DCDBS84 on platelet spreading, stable adhesion, irreversible aggregation and clot retraction, all known to be regulated by outside‐in signaling, similar to RGDS peptide (Figure 4a−d). In addition, the inhibitory effect was dose‐dependent and the efficiency of DCDBS84 at 80 µmol L^−1^ was roughly equivalent to that of RGDS at 1 mmol L^−1^ (Figure 4a−d). However, DCDBS84 did not affect the integrin *β*3 inside‐out signaling‐mediated platelet function which was embodied in soluble fibrinogen binding assay (Figure 4e).

The ischemic events in coronary diseases usually occur in the arteries (3−4 mm in diameter) where the shear rates are low (≈450 s^–1^), which notably increase upon development of stenosis, as high as 64‐fold when stenosis reaches 75%.^[^ ^44^ ^]^ To resist such high shear rates, platelets need to be fully activated.^[^ ^45^ ^]^ 5000 s^–1^ is the maximal shear rate achievable using the flow device available in the present study. The trends we detected in our assays suggest that the inhibitory effect of DCDBS84 on thrombus growth should continue to increase at shear rates higher than 5000 s^–1^. In stenosed coronary arteries, taking a 75% stenosis as example, the shear rates will increase to at least 10000 s^–1^, and stable and firm platelet aggregates are required for thrombus growth under such harsh flow conditions. Whereas in arterioles (0.03 mm in diameter, *γ*_w_ ≈ 1500 s^–1^), smaller and loosely‐packed platelet aggregates, resistant to the lower shear rates, may be able to stop bleeding. We speculate that DCDBS84's minimal disruption of thrombus formation at these shear rates (Figure 4f) may explain its striking ability to preserve hemostatic potential.

Stalker et al. have proposed a hierarchically organized structure for the hemostatic mass in which a “core” of fully‐activated platelets was overlaid by an unstable “shell” of less‐activated platelets.^[^ ^46^ ^]^ Here we propose that by blocking outside‐in signaling, DCDBS84 prevented the transformation of the “core” from the “shell” which were loosely‐packed and only resistant to lower shear rates. The working model of the effects of DCDBS84 on thrombosis and hemostasis was shown in Figure 6.

Proper in vivo animal thrombosis models are required to evaluate the antithrombotic effect of the small molecule. It would be ideal if an animal model of the thrombosis after atherosclerotic plaque rupture is established to mimic the events that exactly occur in cardio‐ and cerebrovascular ischemic patients. There exist the murine plaque rupture models.^[^ ^47^ ^]^ However, it is almost impossible to assess thrombosis at given sites with these models. FeCl_3_‐induced carotid artery thrombosis model is therefore generally used to test the occlusive thrombosis.^[^ ^7^, ^13^, ^14^, ^48^ ^]^

The mainstay of treatment for thrombosis is antiplatelet therapy. Conventional antiplatelet drugs such as aspirin, clopidogrel and eptifibatide (integrilin) were clinically applied at doses by balancing antithrombotic benefit and bleeding risk which were supposed to be insufficient in terms of platelet inhibition.^[^ ^29^, ^30^, ^31^ ^]^ As a matter of fact, in in vivo thrombosis models employed in this study, the effect of these antiplatelet drugs at conventional doses was shown to be far below their maximal antithrombotic potential. Aspirin and clopidogrel at low or maintenance doses (4.3^[^ ^48^ ^]^ or 1.25 mg kg^−1^, respectively) scarcely affected the occlusive thrombus formation with slightly prolong bleeding time and substantial antithrombotic effect was seen at extremely high doses with severe bleeding (Figure 5a,b). As for integrilin, a 0.18 mg kg^−1^ bolus^[^ ^28^ ^]^ was designed taking advantage of its rapid onset of action and short plasma half‐life^[^ ^49^ ^]^ which is commonly used with benefit especially in patients undergoing PCI.^[^ ^50^, ^51^ ^]^ Nevertheless, a drastic antithrombotic effect could be reached by integrilin at an aggressive dose of 16 mg kg^−1^, about 80 times higher than the conventional loading dose after PCI (Figure 5a) which should be closer to an adequate level to ensure an effective prevention of ischemic events. Notably, the antithrombotic effect of DCDBS84 at 10 mg kg^−1^ (Figure 5a), as potent as that by 16 mg kg^−1^ of integrilin, was achieved with unaffected bleeding time (Figure 5b) that was vastly different from the massive bleeding in high dose integrilin‐treated animals. These in vivo data establish that targeting the RT loop of Src SH3 may achieve an adequately potent antithrombotic effect while preserving primary hemostasis.

## 4 Conclusion

In conclusion, a small molecule targeting the RT loop of Src SH3 was identified by its potent antithrombotic effect without increasing bleeding. This newly defined target is thus involved in a mechanism by which thrombosis and hemostasis could be differentially regulated. It seems justified to anticipate that agents targeting this site have potential for facilitating the development of a new class of antithrombotic drugs.

## 5 Experimental Section

The detailed methods are described as follows or in the Supporting Information. Primary data for all studies are provided in Section S1 in the Supporting Information.

### Molecular Docking and Virtual Screening

Molecular docking simulation was used to construct the binding mode of *β*3 heptapeptide (NITYRGT) with SH3 and conduct virtual screening. PyMOL was used to show the comparison of binding modes of *β*3 heptapeptide and PXXP‐containing peptide APPIPPPR with the crystallographic structure of Src (PDB code: 2H8H).^[^ ^52^ ^]^ GLIDE program^[^ ^53^, ^54^ ^]^ (Schrödinger, LLC, New York, NY, 2015) was employed to perform the molecular docking.

### Animals and Reagents

C57BL/6 mice were purchased from Slac Corporation (Shanghai, China). Src^E97A^ transgenic mouse model was generated using gene targeting and blastocyst injection technologies in C57BL/6 mice. Information of the reagents is shown in the Supporting Information.

### Peptide Synthesis

All peptides were purchased from GL Biochem Corporation. Integrilin was purchased from Chinese Peptide Company.

### Plasmid Construction

Plasmids of Flag‐Src^R95A^, Flag‐Src^E97A^, Flag‐Src^G116A^, Flag‐Src^W118A^, and Flag‐Src^Y131A^ were constructed using the QuickChange site‐Directed Mutagenesis Kit (Agilent, USA).

### Establishment of 293T Cell Lines Stably Expressing Wild Type or Mutated Src

293T cells with stable *β*3 (pcDNA3.1(‐)/*β*3) expression was established.^[^ ^55^ ^]^ The plasmids of Flag‐Src^WT^ and mutants (Flag‐Src^R95A^, Flag‐Src^E97A^, Flag‐Src^G116A^, Flag‐Src^W118A^, and Flag‐Src^Y131A^) were transfected into the 293T cells with stable *β*3 expression.

### PXXP‐Containing Peptide (RLP1) Binding to Src SH3

The wells of 96‐well plate were coated with the Flag antibody and then incubated sequentially with the purified Src SH3 wild‐type (WT) or mutants (R95A, E97A, G116A, W118A, and Y131A) with Flag tag, biotin‐RLP1 peptide and HRP‐conjugated streptavidin. The binding was then measured using a spectrophotometer (NanoQuant, Infinite M200, TECAN, Switzerland) at 450 nm.

### Src kinase activity

The Src kinase activity was measured using the CycLex c‐Src Kinase Assay/Inhibitor Screening Kit (MBL, Japan).

### Expression and Purification of Protein

The recombinant Flag‐tagged Src protein (83‐533, human) was cloned into the pFBDM vector and expressed in Sf9 insect cells by Bac to Bac system. Protein was purified by the anti‐Flag affinity resin column and Superdex 200 Increase 10/300 GL column. His‐Src SH3 and GST‐*β*3 cytoplasmic tail fusion proteins were expressed in *Escherichia coli*.

### Hydrogen‐Deuterium Exchange Mass Spectrometry

Recombinant full‐length Src was incubated with or without DCDBS84 at a 1:12.5 (protein:ligand) molar ratio for 16 h at 4 °C. In HDX‐MS experiments, proteins were diluted into similarly buffered D_2_O solvents, causing labile hydrogens on the protein to undergo exchange solvent deuterium. The exchange reaction was then quenched, followed by proteolytic digestion and LC/MS‐based analysis to measure the exchange rate. Percentage change of deuterium uptake at 60 min was mapped onto the crystal models of Src (PDB code: 2SRC).^[^ ^56^ ^]^

### Surface Plasmon Resonance

SPR assay was performed on Biacore T200 instrument (GE Healthcare, USA). Src SH3 was covalently immobilized on a CM5 chip. DCDBS84 was serially diluted and injected at a flow rate of 30 µL min^−1^ for 120 s of association, 120 seconds of dissociation. The *K*d value of DCDBS84 was determined.

### Nuclear Magnetic Resonance

NMR was performed at 25 °C on a four‐channel Bruker Avance III 600 MHz spectrometer with a TCI cryoprobe.

Pull‐down, platelet preparation, co‐immunoprecipitation, platelet spreading, stable adhesion on immobilized fibrinogen, aggregation, fibrin clot retraction, and soluble fibrinogen binding were performed as described previously.^[^ ^12^, ^57^ ^]^

### Thrombus Formation under Flow

Ex vivo flow‐based platelet adhesion assay was performed according to previous studies.^[^ ^58^ ^]^

### FeCl_3_‐Induced Thrombosis

FeCl_3_‐induced thrombosis assay was performed as described previously.^[^ ^13^ ^]^ 6‐ to 8‐week C57BL/6 mice were used. Intravenous injection of different concentrations of DCDBS84, integrilin, or dimethylsulfoxide (DMSO). Carotid arterial thrombosis was induced with a filter paper disc (diameter = 2 mm) that was soaked with 1.2 µL of 7.5% FeCl_3_. Filter paper was removed 3 min later, and then blood flow was monitored with a laser doppler system (Model MA‐0.5VB).

### Tail Bleeding Time

The tails of mice (treated with the same method as FeCl_3_ model) were cut 5 mm from the tip, and bleeding was monitored by blotting with filter paper every 15 s. Bleeding exceeding 15 min was immediately stopped.

### Laser‐Induced Cremaster Artery Thrombosis

Cremaster artery thrombosis was induced as previously described.^[^ ^13^, ^59^ ^]^ Arteriolar wall injury was induced with a micropoint laser ablation system (Photonics Instruments, Germany). Platelet accumulation and the adhesion of activated platelets on the sites of the endothelial injury was visualized using the Olympus BX61 W microscope.

### Statistical Analysis

Data were presented as mean ± standard deviation (SD). Sample size (*n*) was shown in each statistical result. Pairwise comparisons were performed using the Student's *t*‐test and two‐tailed analysis (GraphPad Prism software, Ver 8.0.1, La Jolla, CA, USA), and one‐way analysis of variance (ANOVA) was adopted with normally distributed data assuming equal variances (SPSS software, Ver 18, SPSS Inc., Chicago, IL, USA). *p < 0.05, **p < 0.01.

### Study Approval

All mice were housed in a specific pathogen‐free animal facility at Ruijin Hospital, Shanghai Jiao Tong University School of Medicine. All animal protocols were approved by the Animal Ethical Committee of Shanghai Jiao Tong University School of Medicine. Whole blood was acquired from healthy volunteers with informed consent. The study was approved by the Ethical Committee of Ruijin Hospital, Shanghai Jiao Tong University School of Medicine.

## Conflict of Interest

The authors declare no conflict of interest.

## Author Contributions

J.M., K.Z., Z.L., H.Z. contributed equally to this work. X.X. and C.L. conceived and supervised the project; J.M., K.Z., Z.L., H.Z., B.X., Y.W., J.S., J.L., X.S., H.J., T.L., Y.W., N.Z., Q.M., H.Z., and Z.R. performed experiments. X.X., C.L., J.M., K.Z., Z.L., W.X., and J.W. analyzed data; J.M., K.Z., Z.L., X.X., C.L., and W.X. wrote the paper.

## Supporting information

Supporting Information

Supplemental Movie 1

Supplemental Movie 2

Supplemental Movie 3

## Data Availability

The data that support the findings of this study are available in the supplementary material of this article.
