# Supplementary material for: Targeting the RT loop of Src SH3 in Platelets Prevents Thrombosis without Compromising Hemostasis
Source: Adv Sci (Weinh). 2022 Jan 12;9(7):2103228. doi: 10.1002/advs.202103228 (PMC8895158; doi:10.1002/advs.202103228)
Supplement: Supplementary file 1 — Supporting Information [file ADVS-9-2103228-s001.pdf]

ADVANCED SCIENCE
Open Access

Supporting Information

for *Adv. Sci.*, DOI: 10.1002/advs.202103228

Targeting the RT loop of Src SH3 in platelets prevents thrombosis without compromising hemostasis

Jianhua Mao[1#], Kongkai Zhu[2#], Zhangbiao Long[1#], Huimin Zhang[2,3#], Bing Xiao[1], Wenda Xi[4], Yun Wang[1], Jiansong Huang[1], Jingqiu Liu[2], Xiaofeng Shi[1], Hao Jiang[2], Tian Lu[2], Yi Wen[2], Naixia Zhang[2], Qian Meng[2], Hu Zhou[2], Zheng Ruan[1], Jin Wang[1], Cheng Luo[2,3,5], Xiaodong Xi[1*]*

Supporting Information

Targeting the RT loop of Src SH3 in platelets prevents thrombosis without compromising hemostasis

Short title: Differential regulation of thrombosis/hemostasis

Jianhua Mao[1#], Kongkai Zhu[2#], Zhangbiao Long[1#], Huimin Zhang[2,3#], Bing Xiao[1], Wenda Xi[4], Yun Wang[1], Jiansong Huang[1], Jingqiu Liu[2], Xiaofeng Shi[1], Hao Jiang[2], Tian Lu[2], Yi Wen[2], Naixia Zhang[2], Qian Meng[2], Hu Zhou[2], Zheng Ruan[1], Jin Wang[1], Cheng Luo[2,3,5], Xiaodong Xi[1*]*

[1]State Key Laboratory of Medical Genomics, Shanghai Institute of Hematology, Collaborative Innovation Center of Hematology, Ruijin Hospital, Shanghai Jiao Tong University School of Medicine; Shanghai, 200025, China.

[2]Drug Discovery and Design Center, the Center for Chemical Biology, State Key Laboratory of Drug Research, Shanghai Institute of Materia Medica, Chinese Academy of Sciences, University of Chinese Academy of Sciences; Shanghai, 201203, China.

[3]School of Life Science and Technology, Shanghai Tech University; Shanghai, 201210, China.

[4]Shanghai Institute of Hypertension, Ruijin Hospital, Shanghai Jiao Tong University School of Medicine; Shanghai, 200025, China.

[5]School of Pharmaceutical Science and Technology, Hangzhou Institute for Advanced Study, UCAS; Hangzhou, 310024, China.

[#]These authors contributed equally to this work.

[*]Corresponding Authors: Cheng Luo, Drug Discovery and Design Center, the Center for Chemical Biology, State Key Laboratory of Drug Research, Shanghai Institute of Materia Medica, Chinese Academy of Sciences, 555 Zu Chongzhi Road, Shanghai 201203, China. Phone: +86-21-50806918; Fax: +86-21-50807188; email: cluo@simm.ac.cn or Xiaodong Xi, Shanghai Institute of Hematology, Ruijin Hospital, Shanghai Jiao Tong University School of Medicine, 197 Second Ruijin Road, Shanghai 200025, China. Phone: +86-21-34187218; Fax: +86-21-64743206; email: xixiaodong@shsmu.edu.cn

The PDF file includes:

Supporting Materials and Methods

Figure S1. Screening small molecules by the inhibitory effect on platelet spreading on immobilized fibrinogen and binding affinity for SH3.

Figure S2. Specification of DCDBS84 binding to Src revealed by HDX-MS and NMR spectrum.

Figure S3. The binding capacity of DCDBS84 with mutant Src SH3.

Figure S4. The binding capacity of RLP1 with WT or mutant Src SH3.

Figure S5. The effects of DCDBS84 on Src kinase activity, interaction of RLP1 peptide with Src SH3, interaction of integrin β3 with kindlin 3 and Gα13, and platelet aggregation induced by thrombin.

Figure S6. Supplementary in vivo results of the effects of DCDBS84 and Dasatinib.

Table S1. Small molecule screening process

Legends for Movies S1 to S3

Legend for Data set S1

Legend for 124 compounds information

Other Supporting Materials for this manuscript includes the following:

Movie S1 (.mp4 format). Arteriolar thrombosis: DMSO control.

Intravital video-microscopy of the cremaster muscle arteriolar circulation and platelet thrombus formation (green) after laser injury.

Movie S2 (.mp4 format). Arteriolar thrombosis: DCDBS84 (10 mg/kg) treatment. Intravital videomicroscopy of the cremaster muscle arteriolar circulation and platelet thrombus formation (green) after laser injury.

Movie S3 (.mp4 format). Arteriolar thrombosis: Integrilin (4.2 mg/kg) treatment. Intravital videomicroscopy of the cremaster muscle arteriolar circulation and platelet thrombus formation (green) after laser injury.

Data set S1 (Microsoft Excel format). Raw data.

Information of 124 compounds (Microsoft Excel format).

Supporting Materials

Supporting Materials and Methods

Molecular docking and virtual screening: Molecular docking simulation was used to construct the binding mode of β3 heptapeptide (NITYRGT) with SH3. The full length Src structure (PDB code: 2H8H)[52] was used to compare the binding mode of β3 heptapeptide (NITYRGT) and a class II peptide APPIPPPR with SH3 domain. GLIDE program [53,54] (Schrödinger, LLC, New York, NY, 2015) was employed to perform the molecular docking study. The coordinates of chain A and RGT in the complex crystal structure of SH3: RGT (PDB entry: 4HXJ)[35] were used to construct the docking model. First, NITYRGT peptide was prepared with LigPrep [62] panel to produce multiple output structures with default settings. Then the Protein Preparation Wizard Workflow was used to prepare the protein structures (coordinates of chain A and RGT). And residues located within 15 Å centered on RGT tripeptide in SH3 were defined as NITYRGT binding sites in which the docking grids were created. Finally, the prepared peptide was docked into the binding site with extra precision (XP) mode without any constraint.

In the docking simulation of the SPECS database compounds against SH3, a similar procedure was used as above. Specifically, all of the compounds in SPECS database were prepared with LigPrep panel. Then the prepared compounds were docked into the defined binding sites of SH3 using extra precision (XP) mode without any constraint and were ranked by Glide-gscore. The top-ranked compounds were selected and then subjected to structure cluster. After carefully analysis and comparison, 124 compounds were purchased from SPECS supplier.

Animals and reagents: Src^{E97A} transgenic mouse model was generated using gene targeting and blastocyst injection technologies in C57BL/6 mice. The gene ID is 20779 for the Src Rous sarcoma oncogene sequence. Genotypic identification was performed using PCR and sequencing. Mouse monoclonal antibody SZ-21 to the integrin β3 was a generous gift from C. Ruan (Jiangsu Institute of Hematology, Suzhou, China). Rabbit monoclonal antibody to Src, phospho-Src-Tyr416 (Src^{416}), phospho-Src-Tyr527 (Src^{527}) and horseradish peroxidase- or FITC-conjugated secondary antibodies were purchased from Cell Signaling Technology, anti-His was from Tiangen (Beijing, China), and anti-glutathione-S-transferase (GST),

phospho-β3-Tyr747 (β3^{747}) and phospho-β3-Tyr759 (β3^{759}) were from Abcam. Alex Flour 488-conjugated human fibrinogen was from Sigma-Aldrich. Human α-thrombin was obtained from Enzyme Research Laboratories. Human adenosine diphosphate was purchased from Chronolog. Protein A/G agarose was purchased from Beyotime Biotechnology. All other reagents were obtained from Sigma-Aldrich.

Peptide synthesis: RGT, RGDS and biotinylated RLP1 (RKLPPRPSK)[20] and RLA (GELAAGAAD) peptides were synthesized and purified by GL Biochem Corporation. Integrilin was purchased from Chinese Peptide Company. All these peptides were dissolved in dimethylsulfoxide (DMSO) and stored at -20 °C.

Plasmid construction: Plasmids of Flag-Src^{R95A}, Flag-Src^{E97A}, Flag-Src^{G116A}, Flag-Src^{W118A} and Flag-Src^{Y131A} were constructed using the QuickChange® site-Directed Mutagenesis Kit (Agilent, USA) according to the protocol. Primers were designed by QuickChange® Primer Design Program (Agilent online). The primers used were as follows: Src-SH3 (R95A) Forward: 5’ TCTATGACTATGAGTCTGCG ACGGAGACAGACCTGTC, Reverse: 5’ GACAGGTCTGTCTCCGTCGCAGACTC ATAGTCATAGAG; Src-SH3 (E97A) Forward: 5’ GAAGGACAGGTCTGTCGCCG TCCTAGACTCATA, Reverse: 5’ TATGAGTCTAGGACGGCGACAGACCTGTCCT TC; Src-SH3 (G116A) Forward: 5’ CCAGCCACCAGTCTGCCTCTGTGTTGTTG, Reverse: 5’ CAACAACACAGAGGCAGACTGGTGGCTGG; Src-SH3 (W118A) Forward: 5’ GTGGGCCAGCCACGCGTCTCCCTCTGTG, Reverse: 5’ CACAGAGG GAGACGCGTGGCTGGCCCAC; Src-SH3 (Y131A) Forward: 5’ TAGTTGCTGGG GATGGCGCCTGTCTGTCCTGTG, Reverse: 5’ CACAGGACAGACAGGCGCCAT CCCCAGCAACTAC.

Establishment of 293T cell lines stably expressing wild type or mutated Src: 293T cells were purchased from the American Type Culture Collection (Manassas, VA) and cultured in Dulbecco’s modified Eagle’s medium (DMEM, Invitrogen, USA) with 10% fetal bovine serum (FBS, Moregate, Australia and New Zealand),

penicillin (100 U/mL) and streptomycin (Invitrogen, USA) (100 μg/mL) at 37 °C in a moisturized environment supplied with 5% CO_2. The plasmid ***pcDNA3.1(-)/β3*** was a gift from N. Kiefer (Sino-French Research Center for Life Sciences and Genomics, Ruijin Hospital). [55] 293T stable cell line with persistent integrin β3 expression was established using calcium phosphate cell transfection with ***pcDNA3.1(-)/β3***. Cells were selected using a G418 selection medium and analyzed by flow cytometry using a PE-conjugated anti-human β3 monoclonal antibody. The plasmids of Flag-Src^{WT} and mutants (Flag-Src^{R95A}, Flag-Src^{E97A}, Flag-Src^{G116A}, Flag-Src^{W118A} and Flag-Src^{Y131A}) were transfected into the 293T stable cell line with integrin β3 expression using lipofect-2000 (Invitrogen, USA) according to the protocol provided by the company.

PXXP-containing peptide (RLP1) binding to Src SH3: The wells of 96-well plate were coated with the Flag antibody in coating buffer (0.1 mol/L sodium bicarbonate and carbonate, pH 9.6) and incubated overnight. The next morning, the blocking buffer (0.13 mol/L NaCl, 2.8 mmol/L KCl, 3.2 mmol/L Na_2HPO_4, 1.4 mmol/L KH_2PO_4, 0.05% Tween 20, pH 8.4) was added and incubated for 60 minutes. Then, the wells were incubated sequentially with the purified Src SH3 WT or mutants (R95A, E97A, G116A, W118A and Y131A) with Flag tag, biotin-RLP1 peptide and HRP-conjugated streptavidin. The binding intensity of the biotin-RLP1 was then measured using a spectrophotometer (NanoQuant, Infinite M200, TECAN, Switzerland) at 450 nm. To detect the effects of DCDBS84 on the interaction of RLP1 peptide with Src SH3, DCDBS84 (100 μmol/L) was added into the wells when biotin-RLP1 peptide incubated with Src SH3.

Src kinase activity: The Src kinase activity was measured using the CycLex c-Src Kinase Assay/Inhibitor Screening Kit (MBL, Japan) according to the protocol provided by the company, detailed as follows. In an assay plate with wells pre-coated with “Tyrosine kinase-substrate-1”, 10 μL of Src positive control (0.1 U/μL) or serial dilution of Src positive control were added on ice. Kinase reaction was initiated by adding 90 μL of kinase reaction buffer per well. The plate was then covered with plate sealer and incubated at 30 °C for 30 minutes. After washing the wells five time with wash buffer, residual wash buffer was removed by gentle tapping or aspiration. 100 μL of HRP conjugated detection antibody was then added into each well, followed by sealed incubation at room temperature for 60 minutes and then washing

5 times. After removing the residual wash buffer, 100 μL of substrate reagent was added to each well and then incubated at room temperature for 5-15 minutes. Next, 100 μL of stop solution was added to each well. Absorbance in each well was measured using a spectrophotometric plate reader at 450 nm. To detect the effects of DCDBS84, PP2 and Dasatinib on Src kinase activity, DCDBS84 (20 μmol/L, 40 μmol/L, 80 μmol/L), PP2 (100 μmol/L) or Dasatinib (0.1 μmol/L) was added into the reaction wells together with Src.

Expression and purification of protein: The recombinant Flag-tagged Src protein (83-533, human) with the TEV restriction site was cloned into the pFBDM vector and expressed in Sf9 insect cells by Bac to Bac system (Invitrogen, USA). The cells were infected with the baculoviruses at 27 °C for 48-72 h before collection. Cells were lysed in buffer containing 20 mmol/L HEPES pH 7.4, 150 mmol/L NaCl, 1 mmol/L DTT，1 × protease inhibitor cocktail (Roche, Switzerland) and 1 mmol/L PMSF. Cell lysate supernatants were loaded to column packed with anti-Flag affinity resin (GenScript Corporation, USA), washed by lysis buffer, and finally eluted with 0.2 mg/ml Flag peptide (GenScript Corporation, USA). TEV enzyme was added to digest Flag-tag overnight at 4 °C. The collected samples were concentrated and loaded onto Superdex™ 200 Increase 10/300 GL column (GE Healthcare, USA) for further purification and the buffer was exchanged to 20 mmol/L HEPES pH 7.4, 150 mmol/L NaCl.

His-Src SH3 and GST-β3 cytoplasmic tail fusion proteins were expressed in *E coli* and were grown in LB broth at 37 °C. All these plasmids were verified by DNA sequencing. 200 μmol/L IPTG was added into the cultural medium to induce expression. When the OD (600) value was approximately 0.6-0.8, the fermentation was continued for 12 hours at 22 °C. Then, the bacteria were harvested by centrifugation and lysed with lysis solution. The His-Src-SH3 clear lysate was loaded onto a His-trap column, then elution by buffer containing imidazole. The eluant was cleaved by thrombin overnight at 22 °C, followed by purification using Q column. Finally, the purified Src SH3 protein was obtained. When the His-Src-SH3 was used for pull-down assay, thrombin cleavage was omitted. And GST-β3 cytoplasmic tail fusion protein could be eluted by the buffer containing glutathione.

Hydrogen-deuterium exchange mass spectrometry (HDX-MS): Recombinant full-length Src (20 mmol/L HEPES pH 7.4, 150 mmol/L NaCl) was incubated with

or without compound at a 1:12.5 (protein: ligand) molar ratio for 16 h at 4 °C. HDX exchange was performed on a Fusion Orbitrap mass spectrometer (Thermo Fisher, USA), equipped with a PAL3 autosampler (Leap Technologies, USA) and Ultimate NCS-3500RSLC pump system (Thermo Fisher, USA). The autosampler sample plate was held at 10 °C, where exchange was initiated with addition of D_2O solution (20 mmol/L HEPES pH 7.4, 150 mmol/L NaCl) to protein and ligand samples. The exchange reaction was performed at various time points (10 s, 1 min, 10 min, 20 min and 60 min) before being arrested with addition of cold quenching buffer (1 mol/L guanidine hydrochloride, 25 mmol/L citric acid, 0.1 mol/L TCEP) to a final pH of 2.3. Samples were injected at 50 μL/min across an immobilized VIII / Pepsin Cartridge (2.1 mm × 30 mm, NovaBio Assays) and digested. Peptide fragments were subsequently collected on a Acclaim PepMap300 C18 column (5 μm, 1.0 mm × 15 mm; Thermo Fisher, USA) for desalting with 0.1% formic acid in H_2O and then isolated by liquid chromatography using an ACQUITY UPLC Peptide CSH C18 column (130 Å, 1.7 μm, 1 mm × 50 mm; Waters, USA) at a flow rate of 45 μL/min with an acetonitrile gradient starting with 1% and increasing to 35% over 10 min. Mass spectrometric data were acquired using a Fusion Orbitrap mass spectrometer (Thermo Fisher, USA) with a measured resolving power of 60,000 at 350-1500 m/z. The deuterium uptake data of the peptides was analyzed using HDExaminer (version 2.4) software. A total set of 173 peptides with 84.3% of Src sequence coverage were employed to track deuterium exchange. Based on three experiments of repeated determinations, 5% of the deuteration difference was selected as the threshold to indicate that the peptides had significant deuteration behavior differences in the experiment. Percentage change of deuterium uptake at 60 min was mapped onto the crystal models of Src (PDB code: 2SRC). [56]

Surface plasmon resonance (SPR): SPR assay was performed on Biacore T200 instrument (GE Healthcare, USA) with HBS-EP running buffer (10 mmol/L HEPES, PH 7.4, 3 mmol/L EDTA, 150 mmol/L NaCl, 0.05% surfactant P20). Protein Src SH3 was covalently immobilized on a CM5 chip. Small molecules were serially diluted and injected at a flow rate of 30 μL/min for 120 seconds of association, subsequently 120 seconds of dissociation The *K*d value of small molecules were determined using Biacore T200 evaluation software (GE Healthcare, USA).

Nuclear magnetic resonance (NMR): All NMR experiments were performed at 25 °C on a four-channel Bruker Avance III 600 MHz spectrometer with a TCI cryoprobe. Complete backbone resonance assignments were obtained from ^{15}N-HSQC, HNCACB and HN(CO)CACB spectra using uniformly ^{15}N/^{13}C-labeled Src-SH3 at a final concentration of ~1.3 mmol/L. Interactions between Src SH3 and DCDBS84 were monitored by 2D ^{15}N-HSQC experiments using uniformly ^{15}N-labeled proteins at a concentration of 50 μmol/L and twenty-fold amount of ligand. NMR data were processed using the program NMR Pipe[63] and analyzed with the software CARA or Sparky (Goddard and Kneller, Sparky 3, University of California, San Francisco, USA). Chemical shift perturbation values (average, avg) for ^{15}N and ^{1}H nuclei were derived from equation:

$$\Delta\delta_{avg} = \sqrt{(\Delta\delta_N / 5)^2 + \Delta\delta_H^2}$$

where N and H represent the chemical shift perturbation value of the amide nitrogen and proton, respectively.

Pull-down: Purified GST-β3 protein coupled to glutathione-sepharose 4B beads and His-Src SH3 were incubated overnight at 4 °C under continuous rotation with different concentrations of DCDBS84 (20 μmol/L, 40 μmol/L, 80 μmol/L) or DMSO. Complexes were washed with PBS 5 times. The complexes were analyzed by Western Blot (WB) assay.

Platelet preparation: Whole blood was acquired from healthy volunteers with informed consent. Anticoagulated with 1/10 volume of 3.8% (w/v) trisodium citrate, the blood was centrifuged at 300 g for 10 minutes at 22 °C to obtain platelet-rich plasma (PRP). The platelet concentration in PRP was adjusted to 1-3×10^8 /mL by adding platelet poor plasma. Washed platelets were prepared with blood anticoagulated with 1/4 volume of acid citrate dextrose (ACD, 85 mmol/L trisodium citrate, 21 mmol/L citric acid, and 83 mmol/L dextrose). The PRP was recentrifuged and the pellets were washed with CGS (13 mmol/L trisodium citrate, 120 mmol/L NaCl, 30 mmol/L glucose, pH 6.5) twice, and were resuspended in HEPES-Tyrode's buffer (137 mmol/L NaCl, 2 mmol/L KCl, 12 mmol/L $NaHCO_3$, 0.3 mmol/L NaH_2PO_4, 5.5 mmol/L glucose, 5 mmol/L N-2-hydroxyethylpiperazine -N′-2-ethane sulfonic acid (HEPES), 1 mmol/L $CaCl_2$, 1 mmol/L $MgCl_2$, and 0.1% bovine serum albumin (BSA), pH 7.4) at a final concentration of 1-3×10^8 /mL platelets. The platelet suspensions were rested for 60 minutes before use.

Co-immunoprecipitation and Western blot: Platelets at a concentration of 2×10^8 /mL were incubated with DMSO or DCDBS84 (80 μmol/L) for 60 minutes at 37 °C, then lysed with NP-40 buffer (0.5% NP-40, 50 mmol/L Tris-HCl, pH 7.2, 150 mmol/L NaCl, 1 mmol/L EDTA, 1 mmol/L sodium vanadate, 1 mmol/L of phenylmethysulfonyl fluoride containing the protease inhibitor. Platelet lysis (500 μg protein) were incubated with anti-integrin β3 antibody or normal mouse IgG as control overnight at 4 °C and subsequently incubated with pre-clear protein A/G beads (20 μL) for 2 hours at 4 °C. Complexes were washed by NP-40 buffer 3 times, followed by WB assay to identify the interaction of integrin β3 with Src, Gα13 or Kindlin3. In addition, the platelets were treated with DMSO, DCDBS84 (80 μmol/L) or integrilin (25 μmol/L) for 60 minutes at 37 °C. After that, the platelets were separated into two parts, one as inactive state, and the other as the activated state when thrombin was added into the platelets on platelet aggregation apparatus (APACT4004, Germany). Platelets were lysed by RIPA (50 mmol/L Tris, pH 7.4, 150 mmol/L NaCl, 1% NP-40, 0.5% sodium deoxycholate, 0.1% SDS) with protease inhibitor cocktail and phosphatase inhibitor cocktail. Then the WB was performed using $\beta3^{747}$, $\beta3^{759}$, Src^{416}, and Src^{527} antibodies.

Platelet spreading on immobilized fibrinogen: Washed platelets were resuspended at a concentration of 1×10^8 /mL in Tyrode's buffer, subsequently pre-incubated with DCDBS84 (20 μmol/L, 40 μmol/L, 80 μmol/L), RGDS (1 mmol/L) and DMSO for 60 minutes and then allowed to adhere and spread on fibrinogen-coated (20 μg/mL) coverslips in the wells of 12-well plate at 37 °C for 60 minutes. After washing with PBS 3 times, the adherent platelets were fixed, and stained with TRIFC-labeled phalloidin as previously described. Finally, the spreading shapes of these platelets were observed by a conventional fluorescence microscope (Leica, Germany) with a 60× objective in small molecule screening, and a laser confocal microscope (Leica TCS SP8, Germany) with a 63× objective in DCDBS84 spreading assay. This assay was performed as described previously. [12,57]

Platelet stable adhesion on immobilized fibrinogen: Platelets were washed and incubated with DCDBS84 (20 μmol/L, 40 μmol/L, 80 μmol/L), RGDS (1 mmol/L) and DMSO for 60 minutes. 100 μL platelets were added to the fibrinogen-coated (20 μg/mL) wells of 96-well plate in triplicate and incubated for 60 minutes at 37 °C. Then the wells were washed vigorously with PBS 3 times to remove unstable

adherent platelets. Then, stable adherent platelets were mixed with p-nitrophenyl phosphatase (PNPP) substrate solution (100 mmol/L of sodium acetate, 1% Triton X-100, 3 mg/mL of PNPP) for 60 minutes at 37 °C. The enzyme reaction was stopped with 1 mol/L of NaOH, and the optical density was measured at 405 nm with a microplate reader. [12,57]

Platelet aggregation: Platelet aggregation was performed as previously described.[12,57] PRP was used for diphosphate (ADP) induced platelet aggregation, and washed platelets were used for thrombin induced platelet aggregation. PRP or washed platelet at a concentration of 3×10^8 /mL were pre-incubated in glass vials in the presence or absence of DMSO, DCDBS84 (20 μmol/L, 40 μmol/L, 80 μmol/L), and RGDS (1 mmol/L) for 60 minutes at 37 °C. Then 2 μmol/L ADP or 0.1 U/mL thrombin was added to stimulate aggregation, and aggregate rate was monitored in a lumi-aggregometer (Chrono-Log, USA).

Fibrin clot retraction: Fibrin clot retraction assay was performed according to methods described previously. [12,57] Platelet were washed and incubated with DMSO, DCDBS84 (20 μmol/L, 40 μmol/L, 80 μmol/L), and RGDS (1 mmol/L) for 60 minutes at 37 °C. Fibrinogen was added to a final concentration of 2 mg/mL, then 250 μL platelets were dispensed into siliconized glass tubes. Subsequently fibrin clot retraction was initiated with 1 U/mL thrombin at 37 °C, and monitored by taking photographs every 15 minutes. The clot surface area on the photographs was quantified with the NIH image 1.67e software. Results were expressed as percentage of retraction (% = area t/area $t_0 \times 100\%$).

Soluble fibrinogen binding: Soluble fibrinogen binding assay was performed as described previously. [12,57] Washed platelets were suspended in Tyrode's buffer at 3×10^8 /mL, and then DMSO, DCDBS84 (80 μmol/L) and RGDS (1 mmol/L) were added to the platelets and incubated 30 minutes at 37 °C. Next, the suspension was incubated with or without 20 μmol/L ADP in the presence of 100 μg/mL Alexa Flour 488-labeled fibrinogen for 30 minutes at room temperature in the dark. Platelet bound fluorescence was measured through a flow cytometer (Beckman Coulter, USA). Control was the group without treatment of DCDBS84, RGDS or DMSO. The blank was assessed using platelets treated without ADP.

Thrombus formation under flow: Ex vivo flow-based platelet adhesion assay was performed according to previous studies. [58] In brief, microfluidic channels

(Fluxion Biosciences Inc. USA) were coated with 20 μg/mL collagen incubated at 4 °C overnight. The next morning, the coated channels were blocked with 2% BSA. Whole blood from healthy volunteers was collected with hirudin as an anticoagulant and incubated with integrilin (100 μmol/L), DMSO and DCDBS84 (200 μmol/L) for 60 minutes at 37 °C. Calcein AM (4 μmol/L) was used to label platelets in the whole blood in the dark. The labelled blood was perfused through the micro-channels at wall shear rates of 500, 1500, or 5000 s^{-1} for 5 minutes. Adherent platelet aggregates were monitored using an inverted fluorescent microscope and CCD camera (Nikon Eclipse Ti-s, Japan). The data were analyzed using the Bioflux 200 software (Labtech, USA).

$FeCl_3$-induced thrombosis: $FeCl_3$-induced thrombosis assay was performed as described previously. [13] 6- to 8-week C57BL/6 mice were anaesthetized by intraperitoneal injection of phenobarbital. Intravenous injection of different concentrations of DCDBS84 (2.5 mg/kg, 5 mg/kg or 10 mg/kg), integrilin (0.18 mg/kg, 4.2 mg/kg or 16 mg/kg), DMSO, saline or Dasatinib (5 mg/kg) was performed 15 minutes before assay. Intragastric administration of aspirin (5.4 mg/kg, 125 mg/kg), clopidogrel (1.25 mg/kg, 10 mg/kg, 200 mg/kg) or saline was carried out by using a gavage needle and syringed at 24 h and 2 h prior to the initiation of the carotid artery injury procedure. Carotid arterial thrombosis was induced with a filter paper disc (diameter = 2 mm) that was soaked with 1.2 μL of 7.5% $FeCl_3$. Filter paper was removed 3 minutes later then blood flow was monitored with a laser doppler system (Model MA-0.5VB).

Tail bleeding time: 6- to 8-week C57BL/6 mice were anaesthetized by intraperitoneal injection of phenobarbital. Intravenous injection of integrilin (0.18 mg/kg or 4.2 mg/kg), DCDBS84 (10 mg/kg), DMSO, saline or Dasatinib (5 mg/kg) were performed 15 minutes before the assay. Intragastric administration of aspirin (5.4 mg/kg, 125 mg/kg), clopidogrel (1.25 mg/kg, 10 mg/kg, 200 mg/kg) or saline were carried out at 24 h and 2 h prior to the initiation of the bleeding time procedure. Then tails of mice were cut 5 mm from the tip with a sharp blade, and bleeding was monitored by blotting with filter paper every 15 seconds. Bleeding time was defined as no evidence of rebleeding for 60 seconds. Bleeding exceeding 15 minutes was immediately stopped by applying pressure.

Laser-induced cremaster artery thrombosis: Cremaster artery thrombosis was induced as previously described in male mice aged 6- to 8 week. [13,59] The mice were anesthetized via intraperitoneal injection of pentobarbital and placed on a thermo-controlled blanket (37 °C). The mice were pretreated for at least 15 minutes with integrilin (4.2 mg/kg), DCDBS84 (10 mg/kg), or DMSO by intrajugular injection. Subsequently, the cremaster muscle was exteriorized and superfused with thermo-controlled (37 °C) bicarbonate-buffered saline for the duration of the experiments. The Alexa Fluor 488-labeled anti-mouse CD41 (anti-mouse CD41, BD Pharmingen, USA; Alexa Fluor 488 antibody labeling kit, Invitrogen, USA; CD41 antibody labelling was performed according to the instructions) and Alexa Fluor 647-labeld anti-mouse CD62P (P-selectin) (Biolegend, USA) at the dose of 0.05 mg/kg body weight were injected into the mice via the jugular vein 3 minutes prior to laser injury. Arteriolar wall injury was induced with a micropoint laser ablation system (Photonics Instruments, Germany). Laser-induced thrombi were generated at different sites in the blood vessel, with new sites upstream of the earlier thrombi. Platelet accumulation and the adhesion of activated platelets on the sites of the endothelial injury was visualized using the Olympus BX61 W microscope and a high-speed camera (Hamamatsu C9300, Japan) through an intensifier (Video Scope International). Fluorescence images were captured at 20 frames per second, and data were analyzed using Slidebook v 5.5 (Intelligent Imaging Innovations, USA). The kinetics of platelet accumulation was analyzed by median fluorescence values of the antibodies as a function of time in about 30 thrombi in 3 mice per group. Statistical difference of fluorescence intensity (mean ± SD) and area under the curve (AUC) at selected time points was also determined using Welch's *t* test.

Supporting figures and figure legends

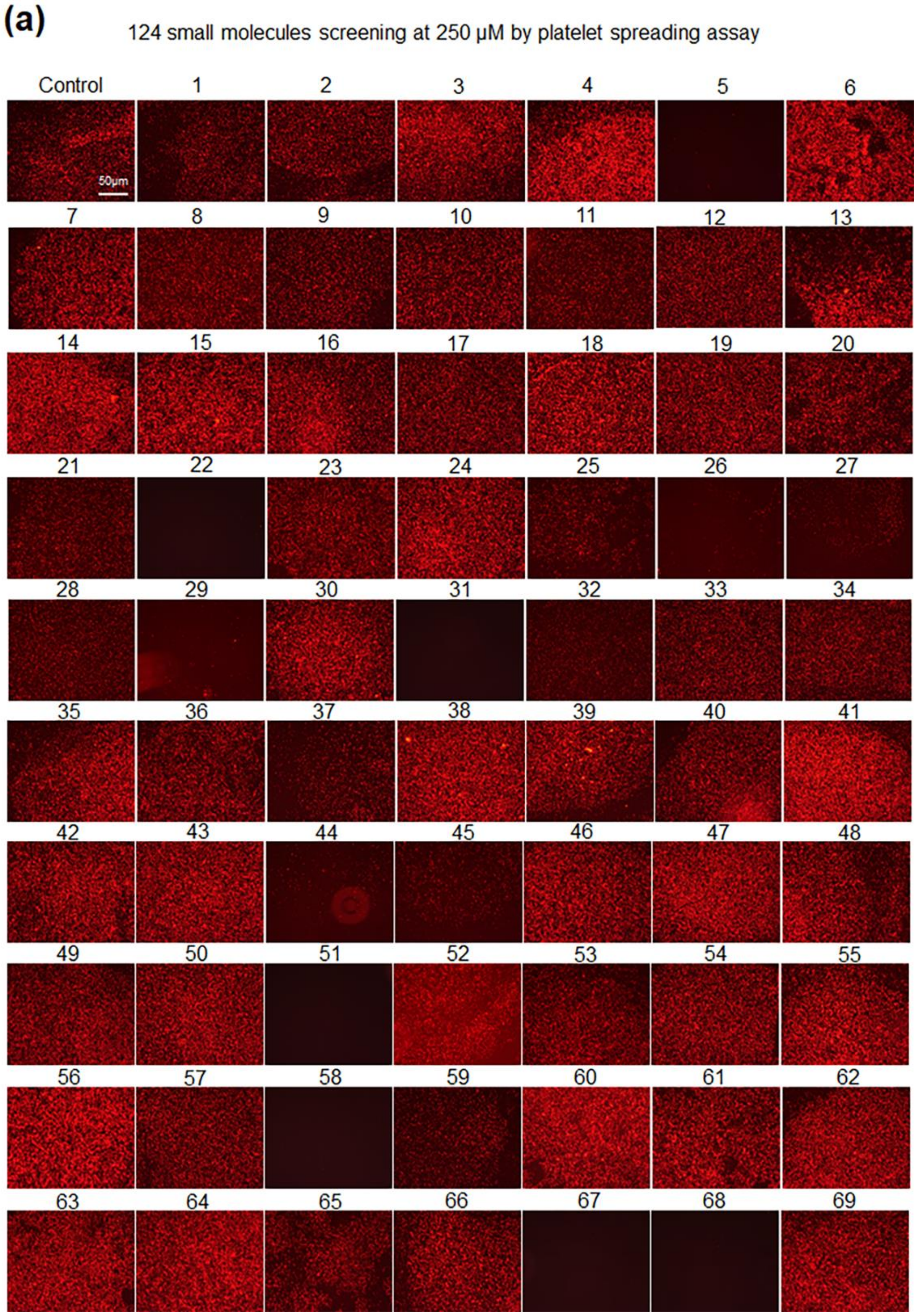

124 small molecules screening at 250 μM by platelet spreading assay

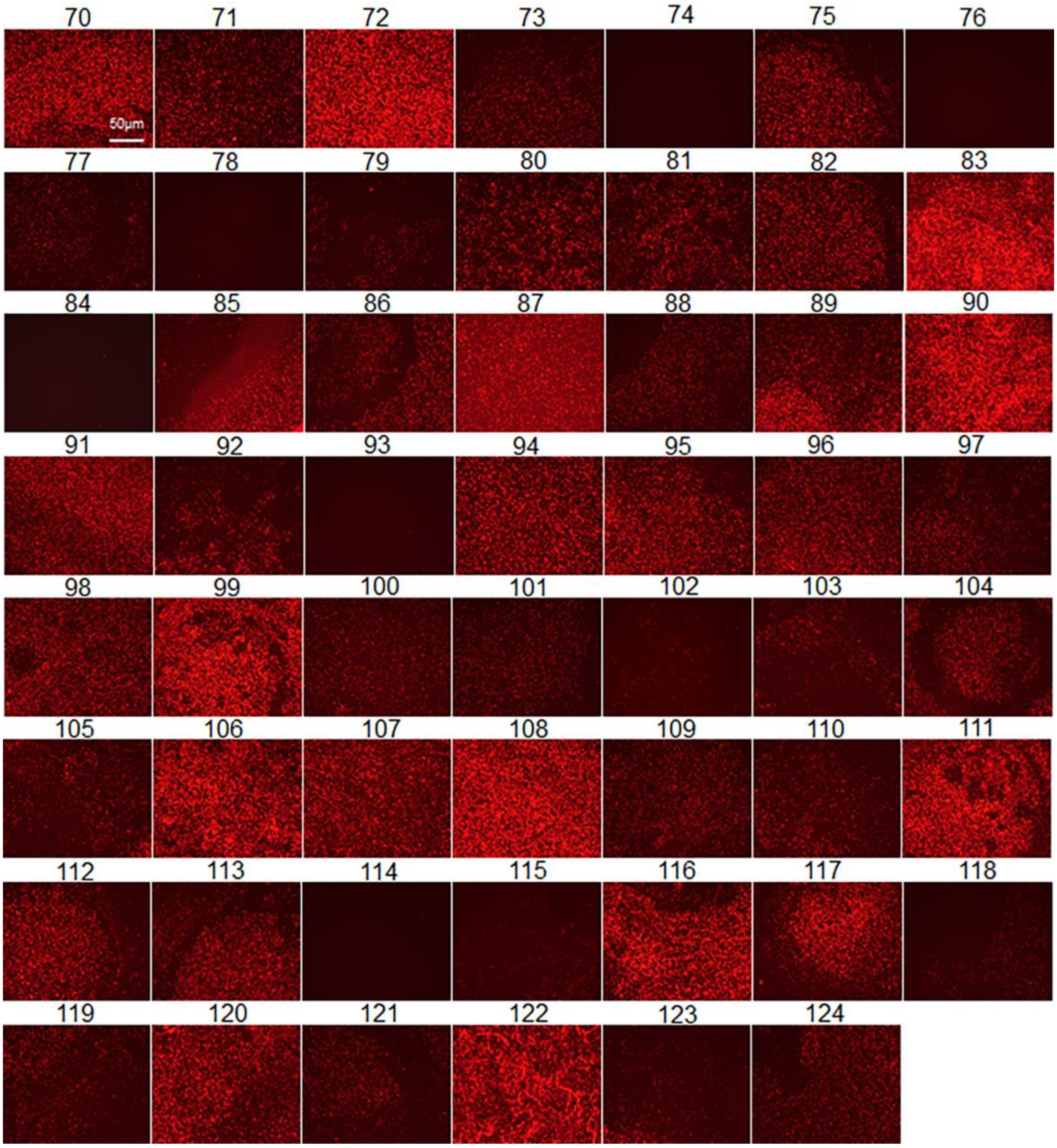

Further screening of 13 small molecules at different concentration by platelet spreading assay

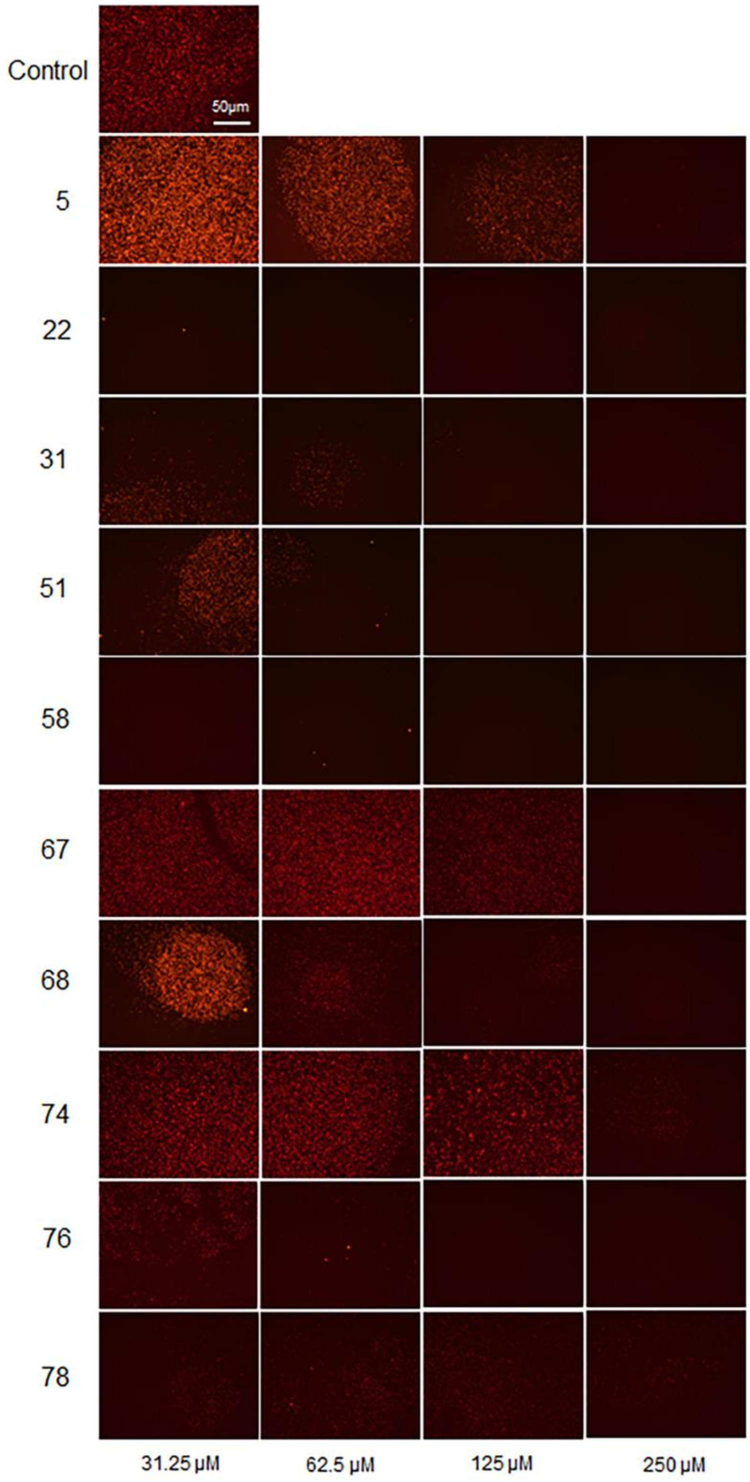

Further screening of 13 small molecules at different concentration by platelet spreading assay

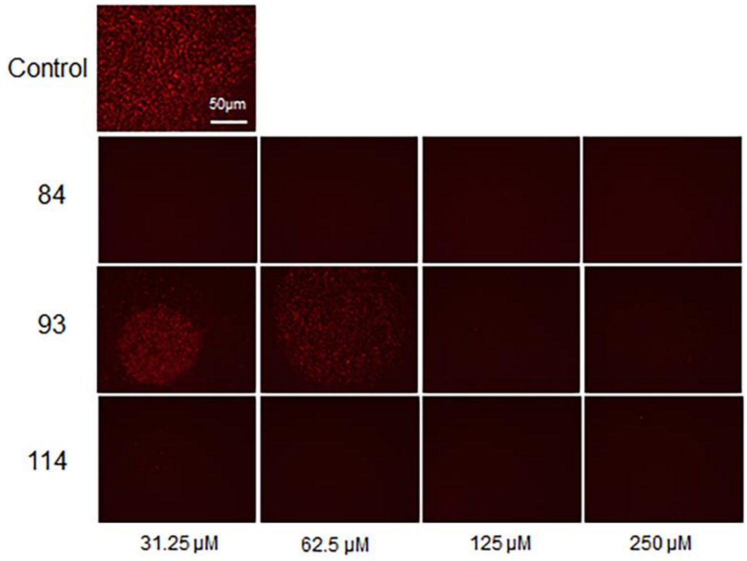

Further screening of 4 small molecules at different concentration by platelet spreading assay

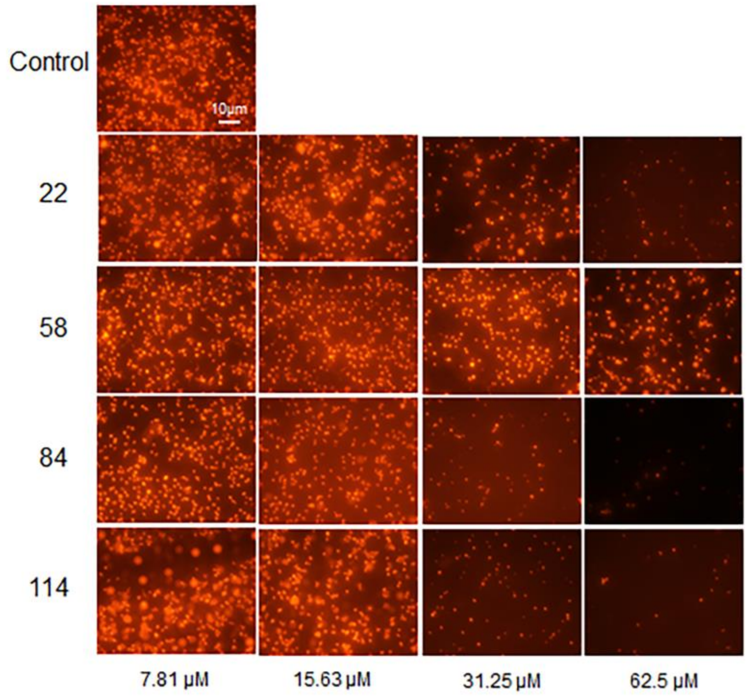

(b)

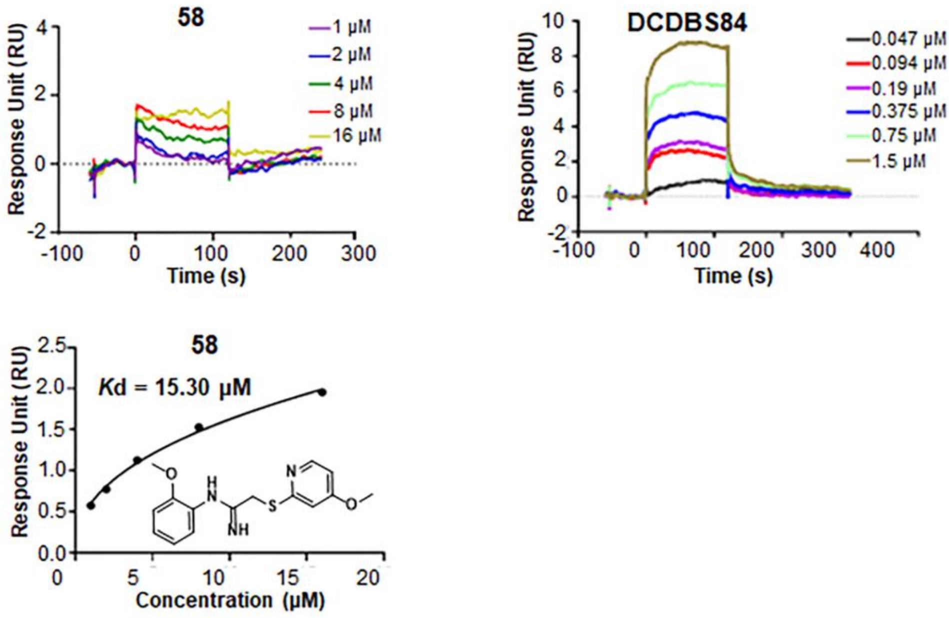

Figure S1. Screening small molecules by the inhibitory effect on platelet spreading on immobilized fibrinogen and binding affinity for SH3

(a) Screening process of 124 small molecules on platelet spreading on immobilized fibrinogen, and in the control group without any agent added. Scale bar is marked in the corresponding picture (50 μm or 10 μm). (b) The SPR results of compounds 58 and 84, the structural formula of 58.

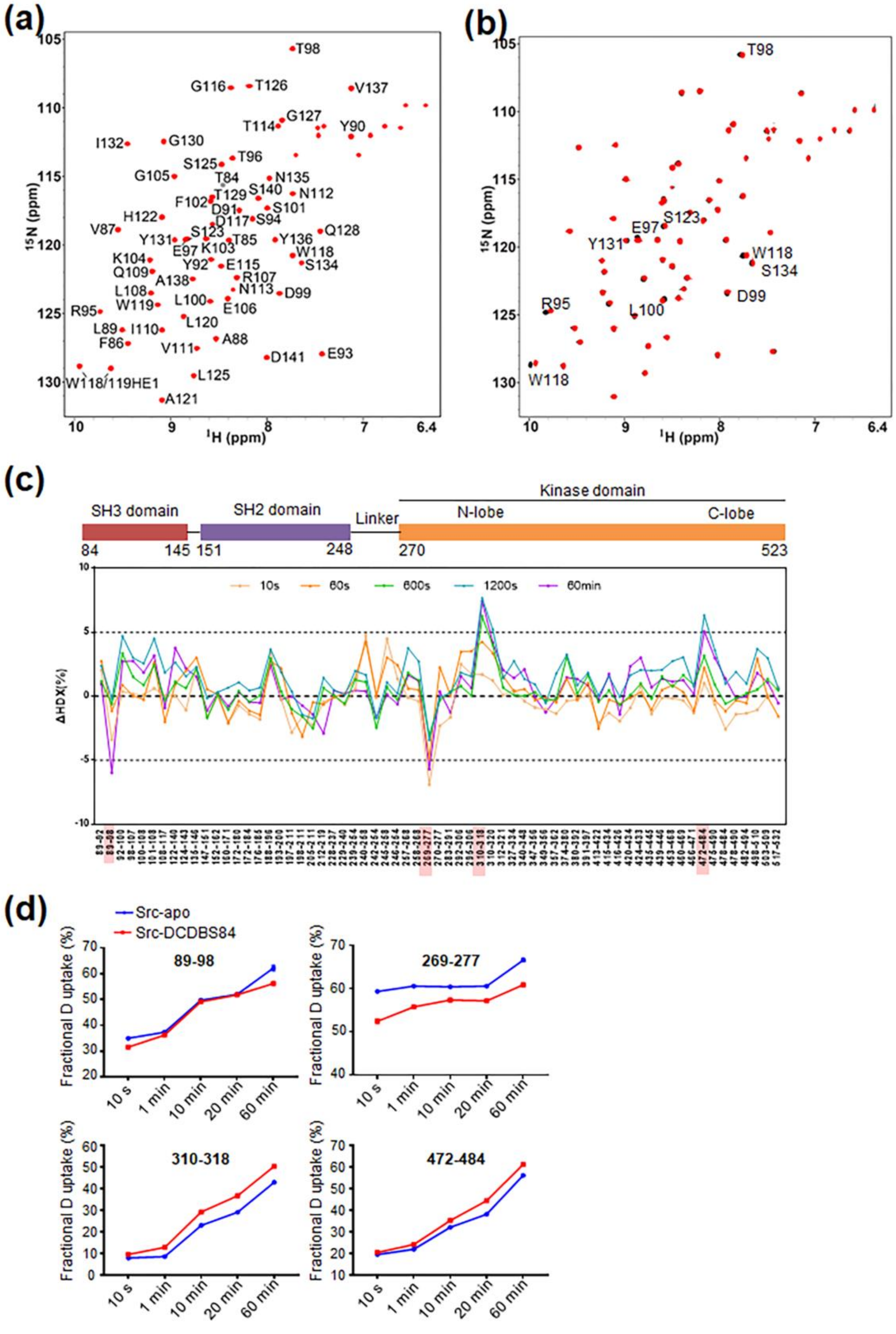

Figure S2. Specification of DCDBS84 binding to Src revealed by HDX-MS and NMR spectrum

(a) Backbone resonance assignments for Src SH3. Amide resonances are labeled with

one-letter amino acid code and the corresponding sequence number. The resonance from residue T84 which is invisible at the current contour level is indicated by a grey dot. The side-chain amide proton resonances from residues W118/119 are also labeled. (b) Overlapped ^{1}H-^{15}N spectrum for Src SH3 in the absence (black) or presence (red) of DCDBS84 at the ratio of 1:20. (c) Deuterium residual plot showing the percentage change of HDX difference for identified peptides from full length Src (86-536, human) upon DCDBS84 combination at different time points, each treatment condition was tested in three independent experiments (n=3). Protein domain architecture of Src is shown on the top. Peptides with significant change of deuterium exchange rates (> 5% decrease or increase) are colored in pink. (d) Deuterium uptake plots at different time points of four identified peptides (fragments 89-98, 269-277, 310-318, 472-484) with significant changes of deuterium exchange rates (> 5% decrease or increase, error bars indicate standard deviations, n=3).

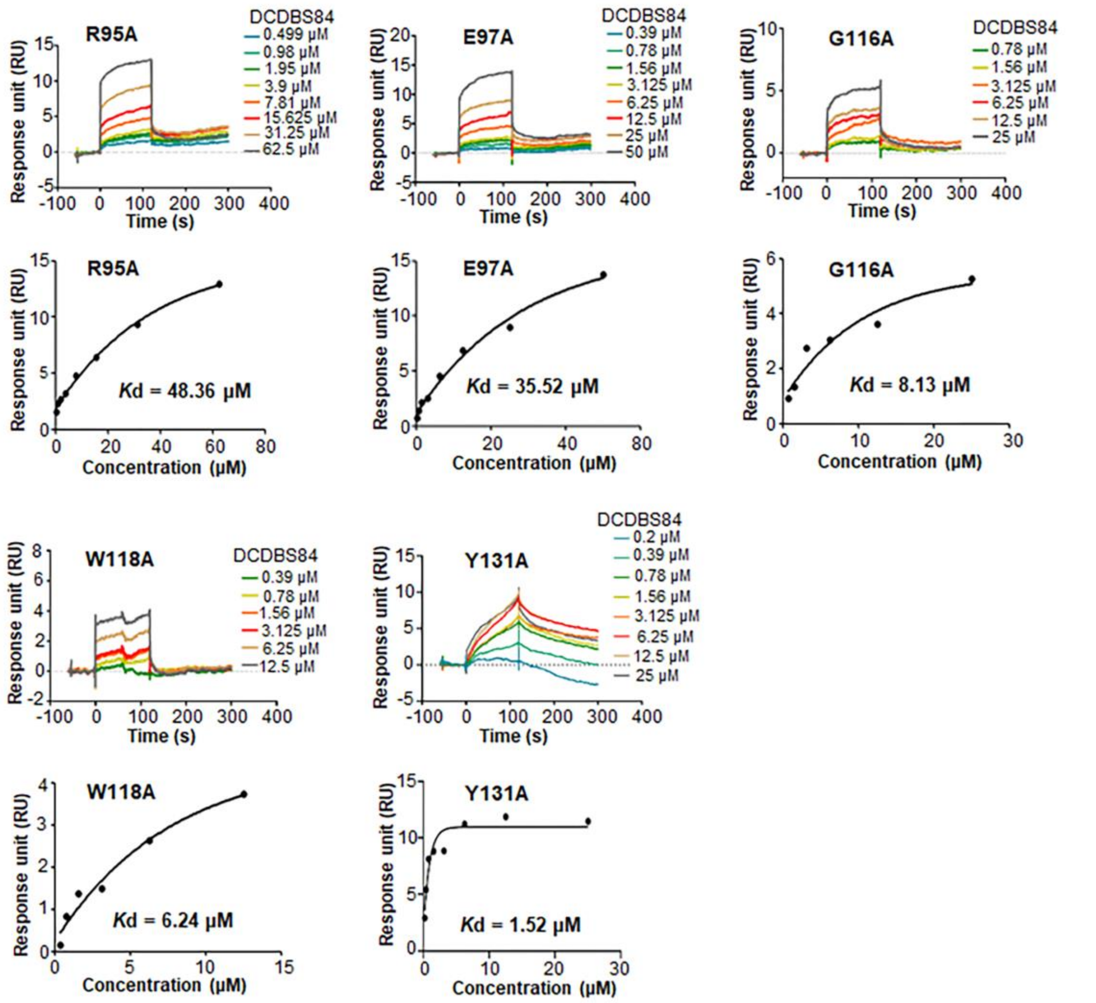

Figure S3. The binding capacity of DCDBS84 with mutant Src SH3

The SPR results of the dissociation constant (*K*d) of DCDBS84 binding to Src SH3 mutants (R95A, E97A, G116A, W118A, and Y131A).

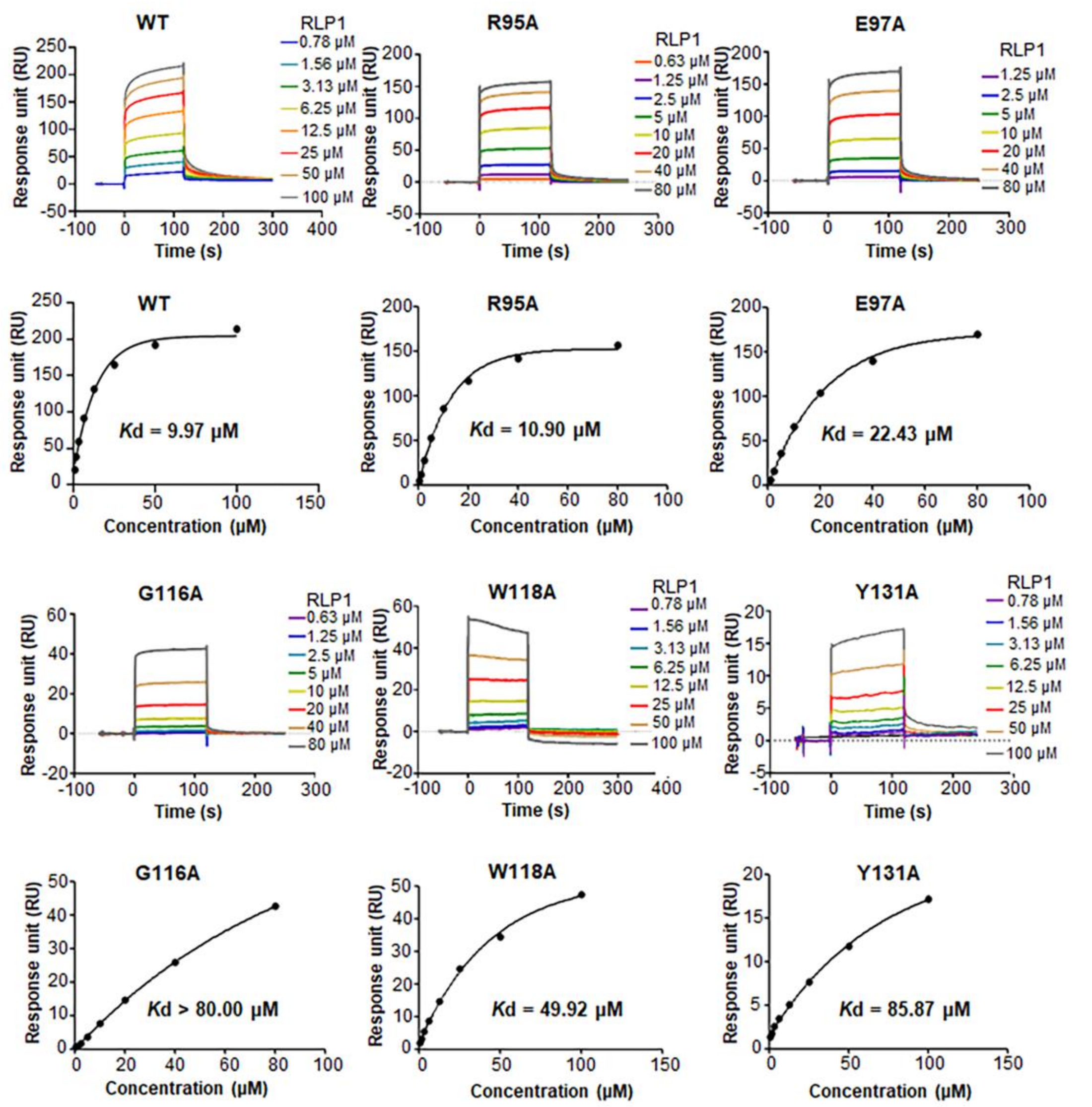

Figure S4. The binding capacity of RPL1 with WT or mutant Src SH3

The SPR results of the dissociation constant (*K*d) of RLP1 peptide binding to Src SH3 mutants (R95A, E97A, G116A, W118A, and Y131A) or WT Src SH3.

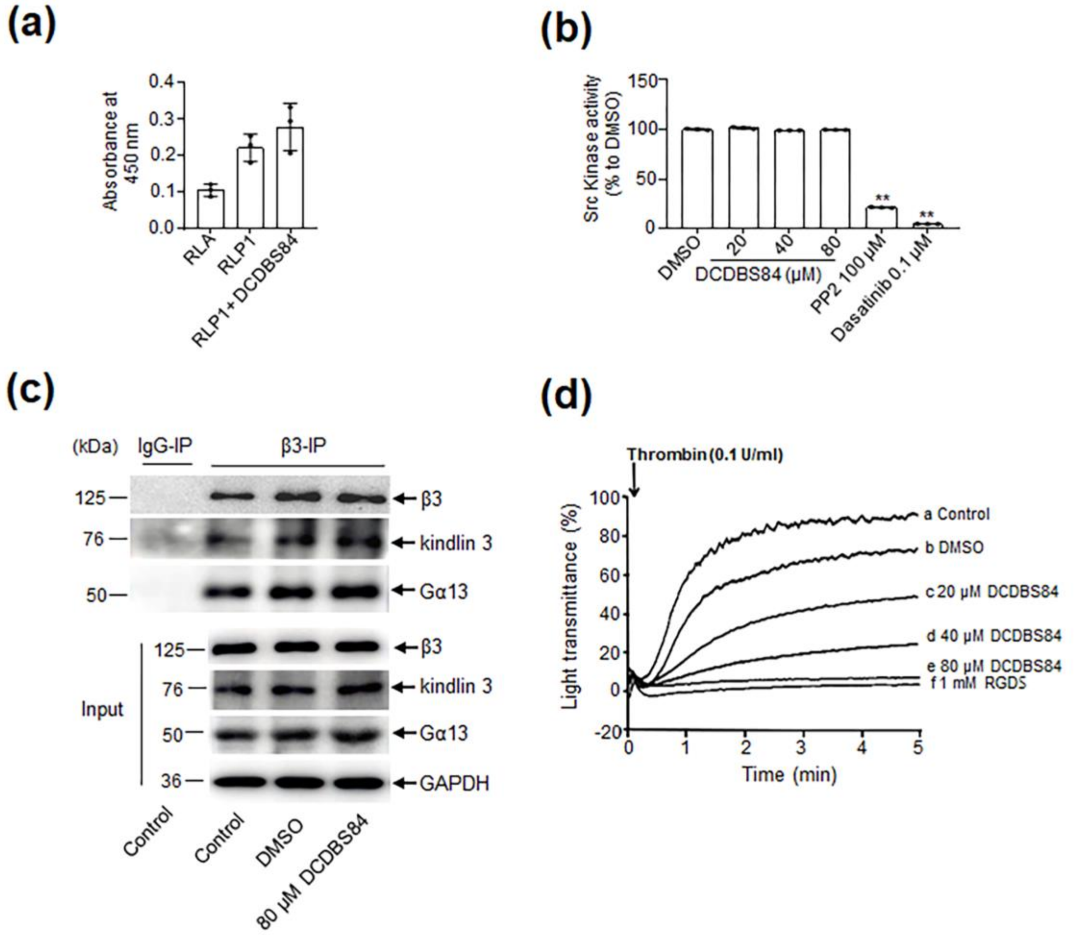

Figure S5. The effects of DCDBS84 on Src kinase activity, interaction of RLP1 peptide with Src SH3, interaction of integrin β3 with kindlin 3 and Gα13, and platelet aggregation induced by thrombin

(a)Effects of DCDBS84 on the interaction of RLP1 peptide with Src SH3 (*n*=3). Data were presented as mean ± SD. Student's *t* test with two-tailed analysis was performed by using GraphPad Prism. (b) Src kinase activity with the treatment of DCDBS84, PP2, Dasatinib or DMSO. Data were shown as mean ± SD. Student's *t* test with two-tailed analysis was performed by using GraphPad Prism (*n*=3, **p<0.01). (c) Platelets lysates were incubated with 80 μmol/L DCDBS84, DMSO or control (without treatment of DCDBS84 or DMSO), immunoprecipitated by anti-integrin β3 antibody and immunoblotted with anti-kindlin 3 and -Gα13 antibodies. (d) Effects of DCDBS84 at different concentrations (20 μmol/L, 40 μmol/L, 80 μmol/L), DMSO, 1 mmol/L RGDS or control (without treatment of DCDBS84, RGDS or DMSO) on platelet aggregation stimulated by thrombin (0.1 U/mL).

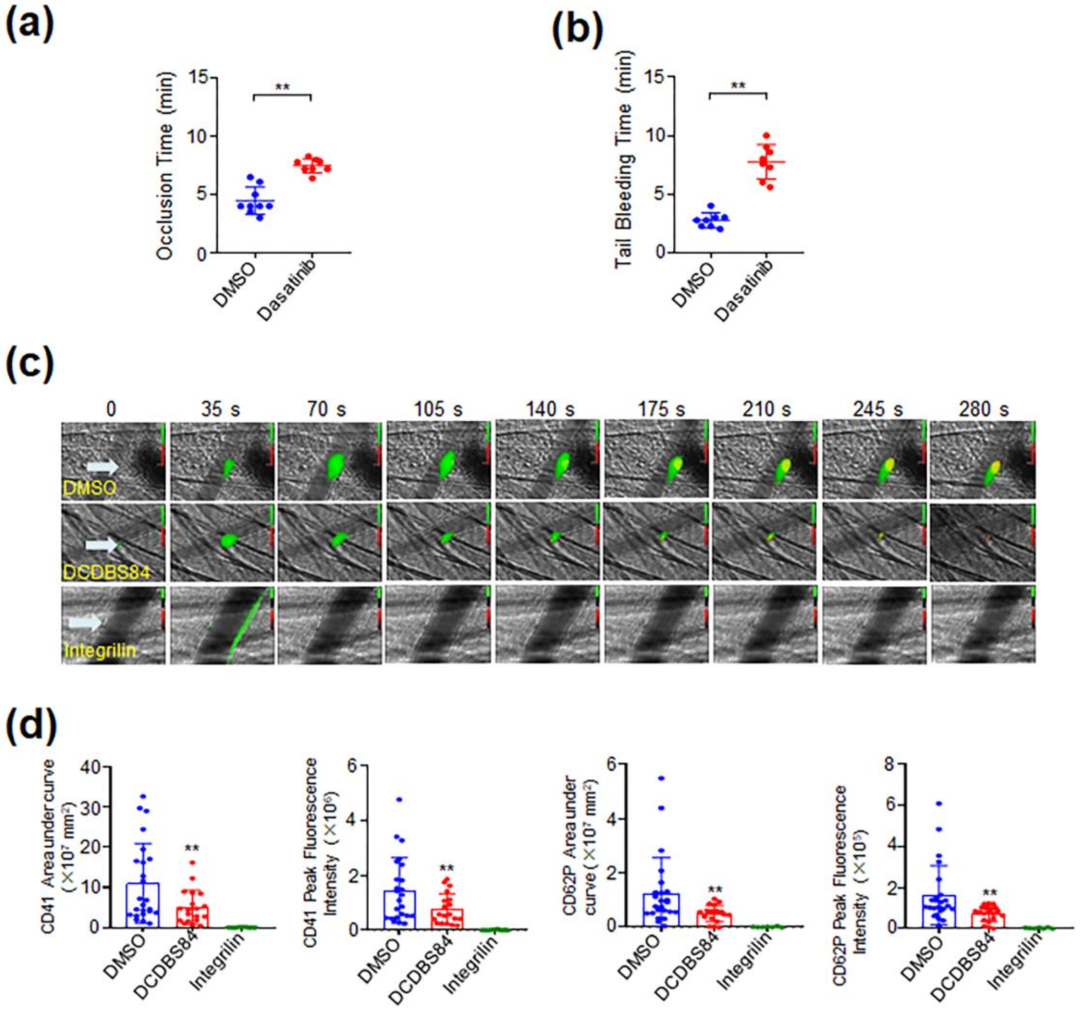

Figure S6. Supplementary in vivo results of the effects of DCDBS84 and Dasatinib

(a) Quantitative analysis of $FeCl_3$-induced occlusive carotid artery thrombosis in mice when treated with Dasatinib (5 mg/kg) or DMSO. Data were presented as mean ± SD. Student's *t* test with two-tailed analysis was performed by using GraphPad Prism (*n*=8, **p<0.01). (b) Tail bleeding time when treated with Dasatinib (5 mg/kg) or DMSO. Data were shown as mean ± SD. Student's *t* test with two-tailed analysis was performed by using GraphPad Prism (*n*=8, **p<0.01). (c) Laser-induced cremaster vessel injury model to monitor the thrombus formation in vivo from 0-280 seconds. (d) Quantitative data to show the CD41 and CD62P fluorescent intensity by area under curve and peak fluorescence. Data were presented as mean ± SD. Student's *t* test with two-tailed analysis was performed by using GraphPad Prism (Sample size (*n*) was showed in each group, from 7 to 24, **p<0.01, compared with DMSO).

Table S1: Small molecule screening process

Screening rounds	Serial number of small molecules
First	1, 2, 3, 4, 5, 6, 7, 8, 9, 10, 11, 12, 13, 14, 15, 16, 17, 18, 19, 20, 21, 22, 23, 24, 25, 26, 27, 28, 29, 30, 31, 32, 33, 34, 35, 36, 37, 38, 39, 40, 41, 42, 43, 44, 45, 46, 47, 48, 49, 50, 51, 52, 53, 54, 55, 56, 57, 58, 59, 60, 61, 62, 63, 64, 65, 66, 67, 68, 69, 70, 71, 72, 73, 74, 75, 76, 77, 78, 79, 80, 81, 82, 83, 84, 85, 86, 87, 88, 89, 90, 91, 92, 93, 94, 95, 96, 97, 98, 99, 100, 101, 102, 103, 104, 105, 106, 107, 108, 109, 110, 111, 112, 113, 114, 115, 116, 117, 118, 119, 120, 121, 122, 123, 124
Second	5, 22, 31, 51, 58, 67, 68, 74, 76, 78, 84, 93, 114
Third	22, 58, 84, 114
Fourth	84

Movie S1. Arteriolar thrombosis: DMSO control. Intravital videomicroscopy of the cremaster muscle arteriolar circulation and platelet thrombus formation (green) after laser injury.

Movie S2. Arteriolar thrombosis: DCDBS84 (10 mg/kg) treatment. Intravital videomicroscopy of the cremaster muscle arteriolar circulation and platelet thrombus formation (green) after laser injury.

Movie S3. Arteriolar thrombosis: Integrilin (4.2 mg/kg) treatment. Intravital videomicroscopy of the cremaster muscle arteriolar circulation and platelet thrombus formation (green) after laser injury.

Data set S1. Raw data (provided as separate excel file).

Information of 124 compounds (provided as separate excel file).
